# Ethnomedicinal plants used by local inhabitants of Jakholi block, Rudraprayag district, western Himalaya, India

**DOI:** 10.1186/s13002-017-0178-3

**Published:** 2017-08-24

**Authors:** Ankit Singh, Mohan C. Nautiyal, Ripu M. Kunwar, Rainer W. Bussmann

**Affiliations:** 1 High Altitude Plant Physiology Research Centre, H.N.B. Garhwal University, Post Box: 14, Srinagar Garhwal, Uttarakhand 246174 India; 2 Practical Solutions, Kathmandu, Nepal; 3 William L. Brown Center, Missouri Botanical Garden, Missouri, USA

**Keywords:** Conservation, Informant consensus factor, Medicinal plants, Sustainable use, Traditional knowledge, Western Himalaya

## Abstract

**Background:**

Ethnomedicinal knowledge of the Indian Himalayas is very interesting because of the wide range of medicinal plants used in traditional medical practice. However, there is a danger of knowledge being lost because the knowledge sharing is very limited and passed on orally. The present study is the first ethnomedicinal study in Jakholi area of Rudraprayag district of Northwestern India. The aim of present study was to identify traditional medicinal plants used by the inhabitants to treat different ailments and document the associated knowledge of these medicinal plants.

**Methods:**

An ethnomedicinal survey was carried out in 72 of 133 villages and alpine pastures of Jakholi block (800–4000 m asl). Door to door surveys and group discussions, applying semi-structured questionnaires were conducted with traditional healers and villagers in local language (Garhwali). Informant Consensus Factor (ICF) was computed to analyse collected ethnomedicinal data.

**Results:**

A total of 78 species (Gymnosperms 3 species, Monocotyledons 12 and 63 Dicotyledons) belonging to 73 genera in 46 families were identified to treat 14 different ailments categories. Most dominant family is Asteraceae (5 species). In disease treated categories, Diseases of the skin (DE) have the highest proportion (29.55%) followed by Gastro- intestinal disorder (GA) (25.89%). The most life form of plants used was herb (56%) followed by tree (23%) while root was the most frequently used part of the plants and the traditional preparation was mainly applied in the form of paste (37%). The highest ICF value (0.99) was found for hair ailments (HA) followed ophthalmologic complaints (OP) and mental afflictions (MA) (0.98).

**Conclusions:**

The present study provides valuable information about traditional knowledge of medicinal plants of Jakholi Block in the Northwestern Himalaya, India. Local communities still possess large traditional knowledge of plants and their therapeutic uses and that the link of that traditional knowledge to modern research could be of importance for the isolation of new phytotherapeutic compounds leading to the development of novel therapeutic active agents. Some of the ethnomedicinal plants are facing high threats and are becoming rare, and conservation initiatives are needed to conserve them for sustainable management in the region.

## Background

The Himalaya is a dynamic area, covering over 18% of the Indian subcontinent and harbouring about 8000 species of angiosperms, 1748 of which are used for their therapeutic properties [1]. The region has been well known for its rich ethnomedicinal flora since ancient times [2].

Plants are used since long time to cure intense chronic diseases, and also as a source of food, shelter and clothing. Due to very low expense and good results these medicinal practices are transmitted through generation to generation and still practiced in different communities. These valuable medicinal plants contain rich bioactive compounds which serve various pharmacological activity. Ethnic people depend on the plants around them to gain economic values and primary health care benefits which is based on need, observation, experience of older ethnic people, and trial and error [3]. About 65% of the Indian population depend on traditional medicine [4]. The study area is interesting due to wide geographic and climatic condition and medicinal plants diversity of Jakholi Block makes this region an especially valuable treasure home of a wide range of wild medicinal and aromatic plants. Ethnic people, shepherd and traditional medicinal practitioner (Vaidyas and Daai) inhabit within a range of 700–3800 m asl and have high knowledge of medicinal plants uses. Local wooden and stone tools are commonly used to prepare medicinal remedies. Most diseases cured by local herbalist are common problems such as respiratory diseases, aches and pains, wounds and musculoskeletal ailments. Inhabitants often use local medicinal plants without prior advice of local traditional healers because they are using these plants since generations. In these connections, the present study was carried out to provide an overview of the knowledge of medicinal plants of the local and traditional healers of Jakholi area and to evaluate the status of these useful medicinal flora for identification of new drugs for health needs and suitable source of income for livelihood of inhabitants. We hypothesize that plant use at Jakholi would show similar response to other Himalayan regions, and that the local medicinal flora would have been overharvested.

The first step of diagnosis by local healers is checking the pulse rate and heartbeat, then examining the forehead, eyes, tongue and in some cases also the urine. The body temperature and colour are major key factors to identify health problems. Medicinal plants play a vital role in the local economy and health care, and demand is increasing. Many populations of medicinal plants seem to drastically decline due to overexploitation and unsustainable harvesting. Most of the important alpine medicinal plants are becoming rare and endangered.

## Methods

### Study area and sites

**Fig.1:**
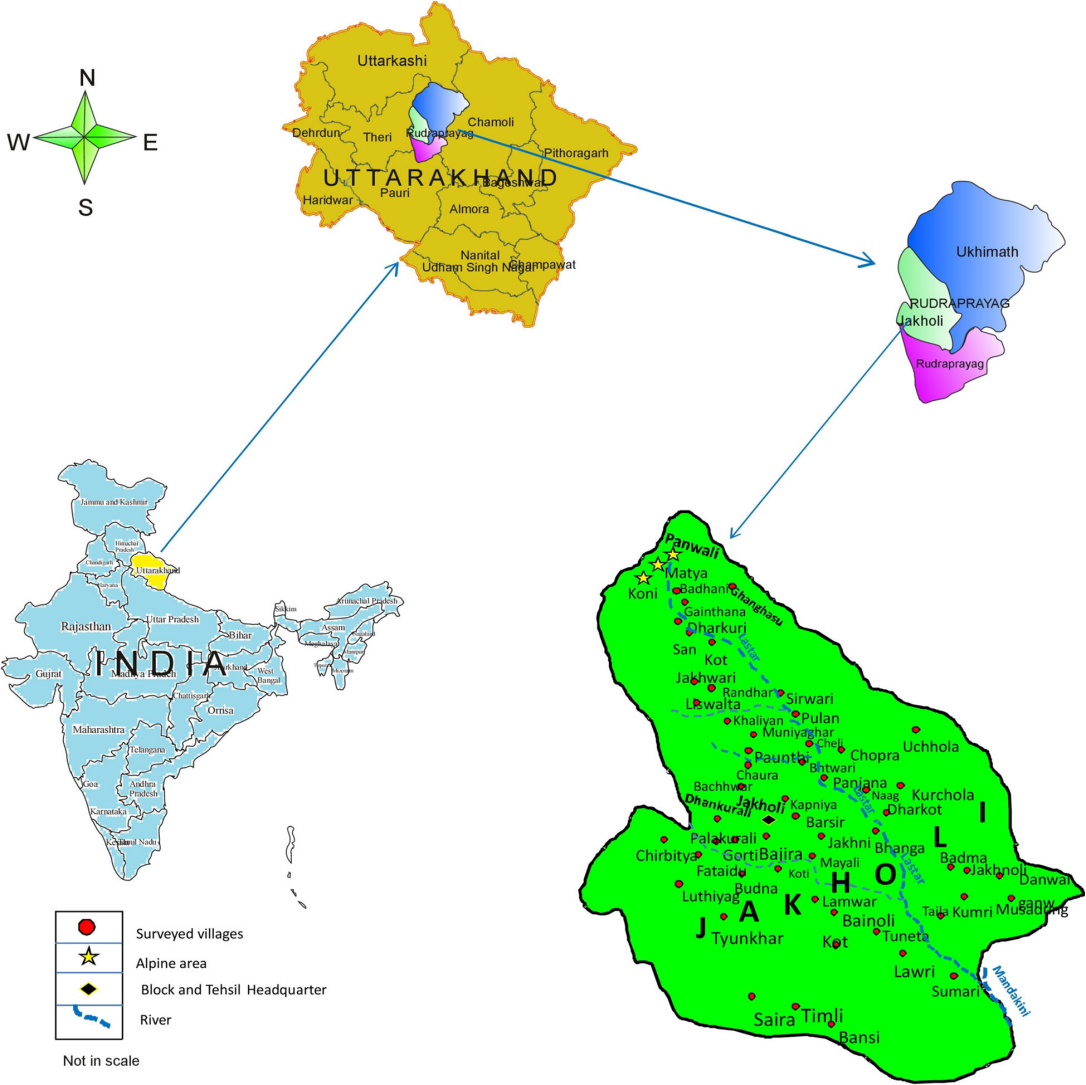
Jakholi Block of district Rudraprayag, Uttarakhand, India

The Jakholi Block is located between the coordinates 30° 37′ 08.88″ to 30° 15′13.47″N and 79° 03′43.79″ to 78° 50′07.97″E (Google Earth Pro Us dept. of State Geographer 2017) in district Rudraprayag western Himalayas India. Medicinal plants sampling was done from alpine meadows of *Panwali Kantha* (3500 – 4000 m) to lower altitudes (800 m) (Fig. 1). Annual average rain fall is around 1850–2000 mm with temperature ranging from − 5 to 15 °C in winter and 20 to 35 °C in summer (High land to lower hills).

**Fig. 2:**
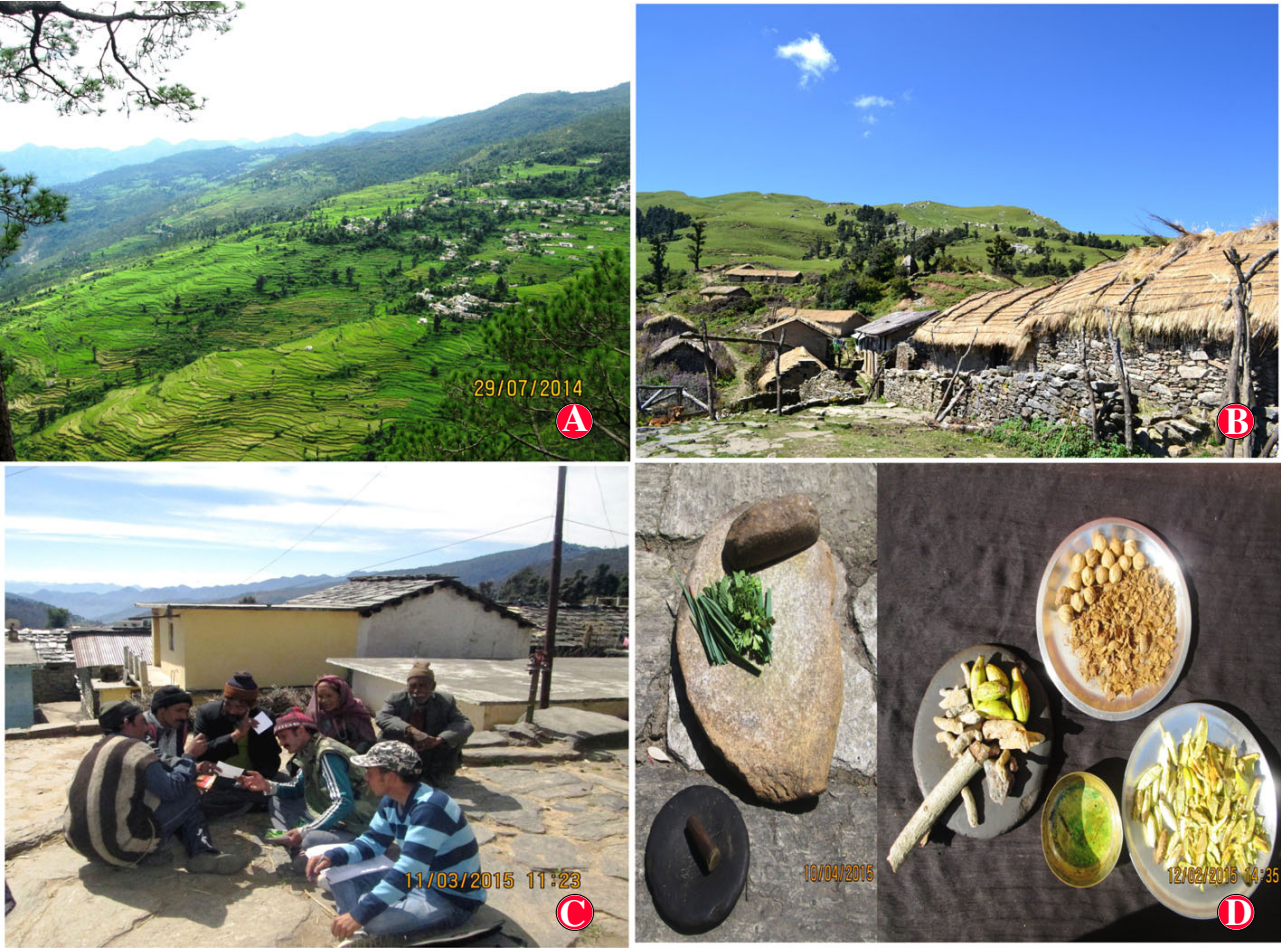
Different localities and collection of information **a** Mountain terrace farming field **b** Panwali kantha homesteads of shepherd **c** Group discussion **d** Traditional formulation with tools

This study was conducted in Jakholi Block of Rudraprayag district, located in north west Uttarakhand. The total area is about 500 km^2^ including 133 villages [5], with a total estimated population of 74,759 (34,126 male and 40,633 female) [6]. Most of the inhabitants live in small villages, and few families are shepherds and stay mostly in alpine areas (Bugyal and Kharka) for 7 – 10 months a year. Most of the inhabitants are farmers. Medical facilities are rare in Jakholi block, and most of the health problems are cured traditionally by local medicine. For chronic diseases people have to travel more than 100 – 200 km from their village to get attention at health facilities. Most of the younger generation, especially men, migrate to cities in order to find employment. Women and elder people live in the villages. Inhabitants are generally belonging to three major cast group, *Jajman, Brahman* and *Oji* (about 65%, 15%, 20% respectively)*,* and Hinduism is the major religion of the inhabitants. Most people speak Garhwali, and Hindi is the secondary major language of the region. Mountain terrace farming is abundant in region, (Fig. 2a), with three crops a year: *Rabi* (October–April/May e.g. Wheat, Barley, Mustard), *Kharif* (April–October e.g. Rice, Corn), and *Jayad* (May–October e.g. Cucumber, Pumpkin, Beans).

### Data collection

A total of 220 individuals were surveyed during the study. Among them some key participants which were experienced and rich knowledge of the medicinal flora were selected for collection and identification of local medicinal plants. All interviews were conducted after obtaining oral and verbal prior informed consents from all individual participants.

The study was conducted during October 2014 to September 2015 in randomly selected villages of Jakholi and information about local medicinal plants was also gathered from shepherds (*Bakrwal*) and ranchers (*Maur*) in the alpine regions, and their homesteads (commonly called Kharka and Maira/Chani viz. *Panwali Kantha, Jadi, Koni and Matya*, Fig. 2b).

Household survey was conducted using individual personal meetings and group discussions as well as field surveys [7–9]. (Fig. 2c). Questionnaires were prepared in English, but interviews were conducted in local language (Garhwali) (Appendix 1) for more convenience and accuracy. As the first author is local person of region so easy understanding and conversations with local people, together more information.

List of local medicinal plants with common name were prepared and photographs were also supplemented for more information about uses and identification. For more reliable information, diseases base questionnaires were used. Information about medicinal plants include local name, plant parts used, drug preparation, mode of administration and doses were recorded. For verification and agreement about the medicinal uses, information given by a respondent was discussed in households as group discussion.

Twenty-five key participants including 11 traditional healers, two shepherds, and 12 other local inhabitants were interviewed and their experience, knowledge of medicinal plants, methods of drug preparation, and practicing with traditional tools (Fig. 2d), etc. were recorded. Monthly schedules were made for data and plant collection including two alpine/pasture surveys were made in July and September. So the participants were interviewed at their homes or at pastures. Medicinal plants were catalogued, and their voucher specimens were collected [10]. Dried specimens were poisoned using 0.1% HgCl_2_ and ethyl-alcohol, and then mounted on herbarium sheets. Collected samples were identified with the help of a local flora [11, 12] and further verified through comparison with prior collections from the botanical survey of India (BSI, Northern circle Herbarium, Dehradun). Plant names were also checked in “Tropicos” http://www.tropicos.org as well as “The Plant List” (http://www.theplantlist.org), and all preserved specimens deposited at the Herbarium of HNB Garhwal University, Srinagar (HAPPRC).

### Data analysis

Data were simply evaluated through informant consensus factor (ICF) described by Trotter and Logan [13, 14] and ethnomedicinal data were checked and compared with previous literature for new use reports. The ICF measures the consensus in using plants in a group about treating an illness in the study area. The ICF was calculated following:

ICF = *Nur - Ntaxa / *(*Nur-1*)

Where Nur refers to the number of use-reports for a particular ailment category and N taxa refers to the number of taxa used for a particular ailment category by all participants. ICF value ranges from 0 to 1. It should be stressed that high ICF value (close to 1.0) indicates that relatively few taxa are used by a large proportion of participants. On the contrary low ICF value (close to 0) indicates a randomly use of plants by participants in treating illness.

Jaccard index (JI) is calculated by comparison of previously published studies from Himalaya and analyzed the percentages of quoted species and their medicinal uses by using the following formula:

JI = *c × 100/a + b - c*

where “a” is the number of species of the area A, “b” is the number of species of the area B, and “c” is the number of species common to A and B [15].

A comparison with previously published data collected from different regions was performed by evaluating percentages of the quoted species and their medicinal uses by applying Sorensen’s similarity index formula [16].

QS = 2c/a + b × 100

where, “a” is number of species in an area A, “b” is number of species in area B and “c” is number of species common to area A and B.

## Results and discussion

### Socio-economy

During the ethnomedicinal survey, a total of 220 people were interviewed, including shepherds at Panwali Kantha (3500 – 4000 m asl), forests and *Kharka* (their homesteads) during June–September 2015. The sociological profile of the participants is given in Table 1. Most participants were from 50 to 59 age group. Only 25 participants were traditional healers (Vaidyas and Daai) and the key informants for this study. Less than 9 % participants were < 40 years old, about 30% were illiterate, while many of the young practitioners hold a degree/diploma (Table 2). Almost all illiterates were > 50 years older.

**Table 1:** Age and gender information of inhabitants and local practitioners

	Gender					
Age group	Male	Female	Vaidyas (male)	Daai (female)	No of persons	Percentage
30 – 39	14	6	0	0	20	9.09
40 – 49	23	14	3	1	41	18.63
50 – 59	27	36	2	4	69	31.36
60 – 69	30	29	3	3	65	29.54
70 – 79	9	7	4	2	22	10
80 +	−	−	3	−	3	1.36
Total	103	92	15	10	220

**Table 2:** Literacy rate of participants

Education level	No. of individuals	Percent
Illiterate	64	29.11
1 - 5^th^	87	39.54
6 - 10^th^	43	19.54
11 - 12^th^	19	8.63
≤ 12^th^	7	3.18
Total	220	

### Ethnomedicinal plants

**Fig. 3:**
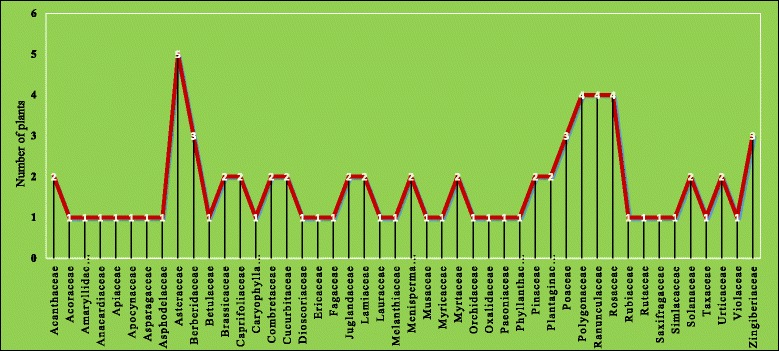
Number of medicinal plants in different families

A total of 78 medicinal plant species belonging to 72 genera of 46 families including 3 gymnosperm species and 75 angiosperms (12 monocotyledons and 63 dicotyledonous) presented in (Table 3) was reported. The most represented families were Asteraceae (5 species), followed by Polygonaceae, Ranunculaceae, Rosaceae (4 species each) and Berberidaceae, Poaceae, Zingiberaceae (3 species each) (Fig. 3). *Picrorhiza kurroa* and *Aconitum heterophyllum* were common ethnomedicinal plants among all participants because these plants are culturally important as they have long been using for generations and due to their rich bioactive constituents.

**Table 3:** Ethnomedicinal plants used by local inhabitants of Jakholi Block, Rudraprayag district, Uttarakhand, India

Plant Family, botanical name and collection number	Common/English name	LF	Parts used	Preparation, Doses, application and ailments categories	∑Citation	Previous uses reported
Acanthaceae						
*Barleria cristata* L. ASR HAPPRC 1461	Kularkatya / Kuladya/Philippine violet	H	Leaves, Root	Leaf and root paste applied in cuts and wounds. (60, DE)	60	1●,2●,3●,4●,5●,6●,7▲, 8●, 9●, 10●, 11▲,12●,13●,14●,15▲,16▲,17●,18●,19▲,20●,21∆,22●,23●,24●,25●,26●,27▲,28●,29●,30▲,31●,32●,33●,34●,35●
*Justica adhatoda* L. ASR HAPPRC 1601	Basingu/Malabar nut	S	Leaves, Stem, Flower	Leaf buds (5–10) decoction (*kwath*) 100 ml a time taken thrice a day for treatment of stomachache and fever (12, 23 GA, FI) Stem used for cleaning teeth. (31,DP) Flower powder (*churna*) used for cough and cold (15, RE) Leaf extract / juice applied for treatment of cut and wounds. (3, DE)	84	1▲,2▲,3●,4●,5∆,6●,7●,8●,9●,10●,11▲12●,13●,14●,15●,16●,17●,18●,19▲,20●,21∆,22●,23▲,24∆,25●,26●,27∆,28●,29●,30●,31∆,32●,33●,34●,35●
Acoraceae						
*Acorus calamus* L. ASR HAPPRC 1456	Bauj / Baj/Sweet flag	H	Rhizome	Rhizome powder (*churna*) (2-4 g) + ½ teaspoon Mishri (Sugar lumps) (2–4 g) gently mixed in cold water (250 ml) drunk thrice a day as it acts as refrigerant. (11, GA) Rhizome powder (*churna*) used for cleaning teeth. (12, DP) Fresh or dried rhizome extract dose of 2–3 teaspoons taken orally thrice a day including 1 taken early morning before eating, for treatment of stomachache (*jonku*). (15, GA) Rhizome garland used to increase child immunity (17, DU) and also used to cure jaundice. (16 GA) Rhizome paste applied in burns, cuts and wounds. (4, DE)	75	1∆,2∆,3●,4∆,5●,6●,7●,8●,9∆,10●,11●,12●,13●,14∆,15●,16●,17●,18∆,19∆,20●,21●,22∆,23▲,24●,25∆,26●,27▲,28●,29●,30∆,31●,32●,33∆,34●,35∆
Amaryllidaceae						
*Allium cepa* L. ASR HAPPRC 1404	Pyaz/Onion	H	Bulb	Bulb juice (*swarasa*) used for treatment of burns, and skin diseases. (69, DE) Bulb juice 1–2 drop is used for earache. (29, EC)	98	1●,2●,3∆,4∆,5●,6●,7●,8●,9●,10●,11●,12∆,13●,14∆,15●,16●,17●,18∆,19●,20∆,21●,22●,23●,24●,25●,26●,27▲,28●,29●,30●,31●,32●,33●,34●,35●
Anacardiaceae						
*Mangifera indica* L. ASR HAPPRC 1618	Aam/Mango	T	Seeds	Seed extract / juice (*rasa*) (Fig. 11) 1 teaspoon used to cure stomachache, dysentery and diarrhea (especially for child) (12,19, GA)	31	1●,2●,3●,4●,5∆,6●,7●,8●,9●,10●,11●,12●,13●,14∆,15●,16●,17∆,18●,19●,20∆,21●,22●,23●,24●,25●,26●,27∆,28●,29●,30●,31●,32●,33●,34●,35●
Apiaceae						
*Centella asiatica* (L.) Urban ASR HAPPRC 1408	Brahmi/Asiatic pennywort	H	Aerial part	*Bramhi* leaf paste applied for treatment of headache. (25, HA) Daily use of *bramhi* juice beneficial for eyesight, leaf powder (*churna*) is also used for same action. (40, OP)	65	1∆,2∆,3▲,4∆,5∆,6●,7●,8●,9∆,10●,11●,12●,13●,14●,15●,16●,17∆,18▲,19●,20●,21●,22∆,23∆,24∆,25∆,26●,27∆,28∆,29●,30●,31●,32●,33●,34∆,35●
Apocynaceae						
*Calotropis gigantea* (L.) Dryand. ASR HAPPRC 1413	Aak/Crown Flower	S	Leaves, Latex	Leaves used for treatment of joint pain, swelling (used as *garam patti*). (37, SK) Latex is useful in skin diseases. (2, DE)	39	1●,2●,3●,4∆,5∆,6●,7●,8●,9●,10●,11∆,12●,13●,14●,15●,16●,17●,18∆,19∆,20∆,21∆,22●,23●,24●,25∆,26●,27∆,28●,29●,30●,31●,32●,33●,34●,35∆
Asphodelaceae						
*Aloe vera* (L.) Brum.f. ASR HAPPRC 1627	Alovera / Gwarpatha	H	Leaves	Leaves sac is used for treatment of skin diseases and burns. (65, DE)	65	1∆,2▲,3●,4●,5●,6●,7●,8●,9●,10●,11●,12●,13●,14●,15●,16●,17●,18●,19●,20●,21●,22●,23●,24●,25●,26●,27∆,28●,29●,30▲,31∆,32●,33●,34●,35●
Asparagaceae						
*Asparagus adscendens* Roxb. ASR HAPPRC 1456	Jhirni/Asparagus	S	Root, Seeds	Root bark (100 g) + Seeds (5-10 g) are ground mixed with *ghee*(clarified butter) (1 tablespoon) and then shade dried; prepared powder (*churna*) is taken 1 teaspoon orally thrice a day with milk to remove weakness. (98, DU) Root (50–60 g) cooked with cow milk (100 ml) (*sodna)* + 1–2 tablespoon sugar, (*paka*) taken orally thrice a day to increase memory power and body weight. Tuberous roots are also galactagogue (increasing and activating mammary gland). (26, GY)	124	1●,2▲,3●,4●,5●,6●,7●,8▲,9●,10●,11●,12●,13●,14●,15●,16●,17●,18∆,19∆,20●,21∆,22●,23●,24●,25●,26●,27∆,28●,29●,30▲,31∆,32●,33●,34●,35●
Asteraceae						
*Eupatorium adenophora* Spreng. Syn-*Ageratina adenophora* (Spreng.) R.M.King & H. Rob. ASR HAPPRC 1529	Basya/Crofton weed	S	Leaves, Stem	Leaves extract / juice applied in cuts and wounds (antiseptic) and burns. (108, DE) Stem piece (7–9 each 10–15 cm) dipped in 500 ml water for a night then this extract is drunk early morning for prompt treatment of pimples. (12, DE) Fresh leaves decoction (*kwath*) is used for treatment of cough and cold (5–10 ml taken orally thrice a day). (18, RE)	138	1●,2▲,3▲,4●,5●,6▲,7●,8●,9▲,10●,11●,12●,13●,14●,15▲,16●,17▲,18●,19●,20●,21●,22●,23●,24●,25●,26●,27▲,28●,29●,30●,31●,32●,33●,34●,35●
*Ageratum conyzoides* (L.) L ASR HAPPRC 1585	Kalabasya / Gundrya/Billygoat-weed	H	Aerial parts	Aerial plant parts extract and paste applied for treatment of burns, cuts and wounds. (36, DE)	36	1▲,2▲,3●,4●,5●,6●,7▲,8●,9●,10●,11▲,12●,13●,14●,15●,16●,17▲,18●,19●,20●,21▲,22●,23●,24●,25●,26●,27∆,28▲,29●,30∆,31●,32●,33●,34▲,35▲
*Jurinea macrocephala* DC. ASR HAPPRC 1620	Bishkandaroo	H	Root	Root paste applied for treatment of boils, pimples, cuts and wounds, and skin diseases. (53,6,30,7 DE)	96	1●,2●,3▲,4●,5●,6▲,7●,8●,9∆,10●,11●,12●,13●,14●,15∆,16●,17●,18●,19●,20●,21●,22∆,23∆,24●,25●,26●,27●,28●,29●,30●,31●,32▲,33∆,34●,35●
*Senecio nudicaulis* Buch-Ham ex D.Don. ASR HAPPRC 1605	Neelbadi	H	Whole plant	Fresh leaves juice (*swarasa*) or extract is used for treatment of ear problem (earache, puss in ear etc.). (10, EC) Whole plants juice with Mishri (Sugar lumps) (4–6 g) used as refrigerant. (21, GA) Leaves juice (1 teaspoon) is used for treatment of stomach problems (*jonku*, mostly occurring in children). (33, GA) 2–3 leaves juice with lukewarm water is used for treatment of fever. (11, FI)	75	1●,2●,3●,4●,5●,6●,7●,8●,9●,10●,11●,12●,13●,14●,15●,16▲,17●,18∆,19●,20●,21●,22●,23●,24●,25●,26●,27▲,28●,29●,30●,31●32●,33●,34●,35●
*Taraxacum officinale* (L.) Syn- *Taraxacum campylodes* G.E. Haglund Weber ex F.H.Wigg. ASR HAPPRC 1434	Kadatu/Common Dandelion	H	Whole plant	Tuberous root paste (*lepa*) applied for treatment of cuts and wounds, headache. (16,17 DE, HA) Root decoction (*kwath*) used for treatment of mouth and throat infection. (2, RE) Whole plant paste (*lepa*) used for skin diseases and boils. (9, DE) Fresh or dried root extract / juice used for treatment of fever. (21, FI)	65	1●,2▲,3●,4●,5∆,6●,7●,8●,9●,10●,11●,12●,13▲,14●,15∆,16●,17●,18●,19∆,20●,21●,22●,23●,24●,25●,26∆,27∆,28∆,29∆,30▲,31●,32●,33∆,34●,35●
Berberidaceae						
*Berberis chitria* Buch. Hamex Lindl ASR HAPPRC 1411	Totar / Totru	S	Root	Decoction (*Rasout*) (Fig. 8) is used for treatment of eye flu and conjunctivitis. (110, OP) Root (5–10 g) rubbed with water then ½ teaspoon taken orally thrice a day for treatment of stomachache. (3, GA) Fresh root extract / juice ½ teaspoon thrice a day for treatment of diabetes. (7, DI)	120	1●,2●,3●,4●,5●,6●,7●,8●,9●,10●,11●,12●,13●,14●,15●,16●,17●,18●,19●,20●,21●,22●,23●,24●,25●,26●,27▲,28▲,29●,30●,31●,32●,33●,34●,35●
*Berberis lyceum* Royle ASR HAPPRC 1594	Kingod/Barberry	S	Root, Inflore-scence	Decoction (*Rasout*) (Fig. 8) of root is used for treatment of conjunctivitis (2–3 drop administered for 3–5 days. (101, OP) ½-1 teaspoon *rasout* taken orally thrice a day for treatment of stomachache. (3, GA) Flower extract / juice is also used for treatment of eye infection. (1, OP) Root is also used in treatment of diabetes. (7, DI)	112	1▲,2▲,3●,4●,5●,6●,7●,8●,9●,10●,11●,12∆,13●,14●,15●,16∆,17●,18●,19●,20●,21●,22●,23●,24●,25●,26●,27▲,28●,29∆,30▲,31▲,32▲,33▲,34●,35●
*Podophyllum hexandrum* Royle Syn- *Sinopodophyllum hexandrum* (Royle) T.S. Ying ASR HAPPRC 1611	Bankakhri/Indian Podophyllum	H	Root	Root paste (*lepa*) used for treatment of cuts and wounds, boils, skin diseases. (3,31,8, DE)	42	1●,2●,3▲,4●,5●,6▲,7●,8●,9▲,10▲,11●,12●,13●,14●,15▲,16●,17●,18●,19●,20●,21●,22∆,23∆,24●,25●,26●,27●,28●,29●,30∆,31∆,32▲,33▲,34●,35▲
Betulaceae						
*Betula utilis* D. Don ASR HAPPRC 1624	Bhoj / Bhojpatra/Himalayan birch	T	Leaves, Bark	Leaf and bark extract / juice is used for treatment of cut and wounds, boils. (17, DE)	17	1●,2●,3∆,4●,5●,6●,7●,8●,9●,10●,11●,12∆,13●,14●,15●,16●,17●,18●,19●,20●,21●,22●,23▲,24●,25∆,26●,27●,28●,29●,30∆,31●,32▲,33▲,34●,35∆
Brassicaceae						
*Brassica juncea (L.) Czern.* ASR HAPPRC 1626	Sarson/Indian mustard	H	Seeds	Seeds oil used as hair tonic and in ear problems. Also used to cure skin diseases (12, 42, 15, HP, EC, DE)	69	1●,2●,3▲,4●,5●,6●,7●,8●,9●,10●,11●,12●,13●,14●,15●,16●,17●,18▲,19●,20●,21●,22●,23●,24●,25●,26●,27●,28●,29●,30●,31●,32●,33●,34∆,35●
*Megacarpaea polyandra* Benth. ex Madden ASR HAPPRC 1616	Barmolu / Barmou	H	Whole plant	Root (4-6 g fresh or dried) rubbed or crushed and mixed with 500 ml water and stayed outside in night covered with cloth and drunk early morning for treatment of fever. (7, FA) Other preparation for fever (*Jar*) and refrigerant: root rubbed in *chonthri* and ½-1 spoon mixed with 1 glass whey / butter-milk (*chanch*) and 1 spoon sugar lumps (Mishri (Sugar lumps)) taken twice a day. Whole plant is refrigerant (cooling effect) (56, GA). Root powder is also beneficial for abdominal problems (17, GA) Root powder also used as antidote of snake bite and scorpion sting (root paste or powder prepared with *ghee* (clarified butter) and applied thrice a day) (9, PB)	89	1●,2●,3▲,4●,5●,6▲,7●,8●,9▲,10●,11●,12∆,13●,14●,15●,16●,17●,18●,19●,20●,21●,22●,23●,24●,25●,26●,27●,28●,29●,30●,31●,32●,33●,34●,35●
Caprifoliaceae						
*Nardostachys jatamansi* (D. Don) DC. ASR HAPPRC 1428	Maasi/Spikenard	H	Rhizome	Rhizome powder ½ teaspoon taken orally thrice a day with water to cure mental disorder and insomnia. (29,35, MA)	64	1●,2●,3▲,4▲,5●,6●,7∆,8●,9∆,10∆,11●,12∆,13●,14●,15●,16●,17●,18●,19●,20●,21●,22∆,23∆,24●,25●,26●,27●,28●,29●,30●,31●,32∆,33●,34●,35●
*Valeriana jatamansi* Jones ASR HAPPRC 1526	Sumaya/Indian Valerian	H	Rhizome	Rhizome powder ½ teaspoon and 5-10 g Mishri (Sugar lumps) taken orally twice a day with lukewarm water for treatment of insomnia (7, MA), abdominal pain, digestive problems (2, GA), cough and cold. (2, RA) Rhizome paste applied in cuts and wounds, boils, skin diseases and headache (4,15,3,2, DE, HA)	35	1●,2▲,3●,4∆,5●,6▲,7●,8●,9▲,10●,11●,12●,13∆,14●,15●,16●,17●,18▲,19●,20●,21●,22∆,23●,24●,25●,26▲,27∆,28▲,29●,30▲,31●,32∆,33▲,34●,35●
Caryophyllaceae						
*Drymaria cordata* (L.) Willd. ex Schult. ASR HAPPRC 1406	Daidya/Tropical Chickweed	H	Aerial part	Paste of aerial part is used to cure herpes (*Makra/Daad*). (6, DE) Leaves juice is used for treatment of fever and headache. (13, FI, HA)	19	1●,2●,3●,4●,5●,6●,7●,8●,9●,10●,11∆,12●,13●,14●,15●,16●,17●,18●,19●,20●,21●,22●,23●,24●,25●,26●,27∆,28●,29●,30●,31●,32●,33∆,34∆,35●
Combretaceae						
*Terminalia bellirica* (Gaertn.) Roxb. ASR HAPPRC 1582	Baheda/Beleric	T	Fruit	Fruit peel powder is useful in cough and respiratory diseases. (22,10, RE)	32	1▲,2▲,3●,4●,5●,6●,7●,8●,9●,10●,11▲,12●,13●,14▲,15●,16●,17●,18∆,19●,20●,21●,22●,23●,24●,25●,26●,27∆,28●,29●,30∆,31●,32●,33●,34●,35●
*Terminalia chebula* Retz. ASR HAPPRC 1598	Haida/Myrobalan	T	Fruit	Fruit dipped in cow urine for 1 week, and then dried in partial shade and stored in jam bottle. ½-1 teaspoon taken orally thrice a day for treatment of cough. (42, RE) Fruit peel rubbed with mustard oil is applied for treatment of skin diseases. (7, DE)	49	1▲,2▲,3●,4●,5●,6∆,7●,8●,9●,10●,11▲,12●,13●,14●,15●,16●,1718∆,19●,20●,21●,22●,23●,24∆,25●,26●,27∆,28●,29●,30▲,31●,32●,33●,34∆,35●
Cucurbitaceae						
*Cucumis sativus* L. ASR HAPPRC 1414	Kakhdi/Cucumber	Cl	Seeds	Seeds (5–10) rubbed with water and 2 teaspoon of the prepared juice (*swarasa*) is given to child twice a day for treatment of fever (*taap*). Massages through juice / swarasa on whole body as refrigerant in fever (*taap)*. (65, FI)	65	1●,2∆,3●,4●,5∆,6●,7●,8●,9●,10●,11●,12●,13●,14●,15●,16●,17●,18●,19●,20●,21●,22●,23●,24●,25●,26●,27∆,28●,29●,30●,31●,32●,33●,34●,35●
*Trichosanthes tricuspidata* Lour. ASR HAPPRC 1599	Yaladu	Cl	Fruit, Seeds	Extract / juice (*swarasa*) of skin / peel of *yaladu* fruit ½-1 teaspoon taken orally thrice a day as refrigerant. (31, GA) Seed powder (*churna*) (½-1teaspoon) taken orally thrice a day for treatment of internal injury. (11, DU)	42	1●,2●,3●,4∆,5∆,6●,7●,8●,9●,10●,11●,12●,13●,14●,15●,16●,17●,18●,19●,20●,21●,22∆,23●,24●,25●,26●,27∆,28●,29●,30●,31●,32●,33●,34●,35●
Dioscoriaceae						
*Dioscorea bulbifera* L. ASR HAPPRC 1552	Genthi/Air Yam	Cl	Tuber	Tuber powder (*churna*) ½-1 teaspoon taken orally thrice a day for curing fever. (17, FI) Tuber paste (*lepa*) applied for treatment of boils. (16, DE)	33	1∆,2∆,3●,4●,5●,6●,7●,8●,9●,10●,11●,12●,13●,14●,15●,16●,17∆,18●,19∆,20●,21∆,22●,23∆,24●,25●,26●,27∆,28●,29●,30●,31●,32●,33●,34●,35●
Ericaceae						
*Lyonia ovalifolia* (Wall.) Drude ASR HAPPRC 1520	Anyar	T	Leaves, Bark	Leaves (4–5) and bark (5–10 g) crushed with 10–20 ml water, prepared in a semi-dried (*avleha*) preparation (*anyarkutu*) applied to cure boils, skin diseases (antiallergic). (33,10, DE)	43	1●,2▲,3●,4●,5●,6●,7●,8●,9●,10●,11∆,12●,13▲,14●,15▲,16▲,17●,18●,19●,20●,21●,22▲,23▲,24●,25●,26∆,27∆,28▲,29●,30▲,31●,32●,33●,34●,35●
Fagaceae						
*Quercus leucotrichophora A. Camus* Syn- *Quercus oblongata* D. Don ASR HAPPRC 1393	Baanj/Himalayan oak	T	Gum, Root, Leaves, Bark	Gum/resin rubbed in *chonthri* then 0.5–1 g given orally thrice a day with lukewarm water for treatment of especially child fever, stomach ache, laxative and refrigerant. (15, 13,36,59 FI, GA) Gum/resin is also used in *stri roga* (female genital disorder, leukorrhea,). (2, GY) Bark extract / juice (½-1 teaspoon) taken orally thrice a day with lukewarm water for treatment of stomachache and abdominal problem. (2, GA)	127	1●,2▲,3●,4●,5●,6●,7●,8●,9●,10●,11▲,12∆,13●,14●,15●,16▲,17●,18∆,19●,20●,21●,22▲,23●,24●,25∆,26▲,27▲,28▲,29●,30∆,31●,32●,33●,34●,35●
Juglandaceae						
*Engelhardtia spicata* Lechen ex Blume ASR HAPPRC 2798	Bish mahua	T	Whole plant	Branches stem and root are used as toothbrush (cleansing teeth) and helpful to remove pyorrhea. (37, DP) Leaves, bark and root paste applied for treatment of boils, cuts and wounds. (50, DE)	87	1●,2●,3●,4●,5●,6∆,7●,8●,9●,10●,11●,12●,13●,14●,15●,16●,17●,18●,19●,20●,21●,22●,23●,24●,25●,26●,27∆,28●,29●,30●,31●,32●,33●,34●,35●
*Juglans regia* L. ASR HAPPRC 1581	Akhor/walnut	T	Whole plant	Leaves, stem or branches, root, used for cleaning teeth and for treatment of pyorrhoea and for shining teeth. (89, DP) Fruit peel paste is used for treatment of tinea pedis (*kaaden*) and boils, cuts and wounds and skin diseases. (28, DE) Bark and leaves paste is applied for skin diseases, cuts and wounds. (9, DE)	126	1●,2▲,3▲,4●,5●,6▲,7●,8●,9▲,10●,11●,12∆,13●,14●,15▲,16∆,17●,18▲,19●,20●,21●,22●,23▲,24●,25∆,26∆,27∆,28●,29●,30▲,31●,32∆,33∆,34●,35●
Lamiaceae						
*Ajuga parviflora* Benth. ASR HAPPRC 1573	Neelkanthi/Small-Flowered Bugleweed	H	Aerial part	Leaves crushed and mixed with water, then the mixture filtered through cloth. This preparation of extract / juice (*swarasa*) in dose of ½-1 teaspoon taken orally thrice a day with 250 ml water is used for treatment of abdominal problems, and also act as refrigerant (cooling effect) (29, GA). Leaves paste prepared with mustard oil applied for treatment of skin diseases, boils, and pimples (6, DE). Fresh aerial part extract / juice (*sawarasa*) 1–2 drop thrice a day for treatment of earache / ear infection (puss in ear) (9, EC).	44	1●,2●,3●,4●,5●,6●,7●,8●,9●,10●,11●,12●,13●,14●,15●,16●,17●,18●,19●,20●,21●,22▲,23●,24●,25●,26●,27●28●,29●,330▲,31▲,32∆,33●,34●,35∆
*Mentha × piperita* L. ASR HAPPRC 1591	Pudina/Peppermint	H	Aerial part	Leaves powder (1 teaspoon) taken thrice a day with lukewarm water acts as appetizer (increasing digestion and hunger) (21, GA). Fresh aerial plant part (2–4 g) + water + ½-1 *kaagji* fruit juice (*Citrus aurantifolia* (Christm.) Swingle) taken once a day acts as refrigerant (cooling effect), carminative (releases intestinal gases or flatulence) (12, GA). Aerial part paste applied for treatment of burns (3, DE).	36	1●,2●,3●,4●,5∆,6●,7●,8●,9●,10●,11●,12●,13●,14●,15●,16●,17●,18∆,19●,20●,21●,22●,23●,24●,25●,26●,27▲,28●,29●,30∆,31●,32●,33●,34●,35●
Lauraceae						
*Cinnamomum tamala* (Buch.-Ham.) T. Nees & Eberm. ASR HAPPRC 1505	Khikoda / Khikhaidu/Indian Bay Leaf	T	Bark, Leaves	Bark powder is used to cure heart diseases (22, DU). ½-1 teaspoon bark powder taken orally thrice a day for treatment of stomachache. (25, GA)	47	1●,2▲,3●,4●,5●,6∆,7●,8●,9∆,10●,11●,12●,13●,14∆,15●,16●,17●,18∆,19●,20●,21●,22●,23∆,24●,25▲,26●,27∆,28∆,29●,30●,31∆,32▲,33●,34●,35∆
Melanthiaceae						
*Paris polyphylla* Sm. ASR HAPPRC1612	Dudhiya / Sankhjadi / Satwa / Myanaru/Himalayan Paris	H	Leaves, Rhizome	Rhizome paste (*lepa*) applied in treatment of cuts and wounds, leaf also used as vegetable and its act as tonic. (36, 1, DE, DU)	37	1●,2●,3●,4●,5●,6●,7●,8●,9∆,10●,11●,12●,13●,14●,15●,16●,17●,18∆,19●,20●,21●,22●,23∆,24●,25●,26●,27∆,28∆,29●,30●,31●,32∆,33▲,34●,35●
Menispermaceae						
*Stephania elegans Hook. f. & Thomson*ASR HAPPRC 1407	Pahari	Cl	Aerial part	Leaf paste applied for treatment of headache. (4, HA) Aerial part (1–2 ft bearing 6–8 leaves) + Mishri (Sugar lumps) (10–15 g) are crushed and dipped in water (500 ml) for a night, then taken as drink in early morning, as it acts as refrigerant. (15, GA) Leaf (4–5) extract ½-1 teaspoon taken orally thrice a day for treatment of fever. (4, FI)	23	1●,2●,3●,4●,5∆,6●,7●,8●,9∆,10●,11●,12●,13●,14●,15●,16●,17●18●,19●,20●,21●,22●,23●,24●,25●,26●,27●,28●,29●,30●,31●,32●,33●,34●,35●
*Tinospora cordifolia* (Willd.) Miers Syn- *Tinospora sinensis* (Lour.) Merr. ASR HAPPRC 1608	Giley/Heart-leaved moonseed	Cl	Whole plant	Aerial part extract / juice is used as refrigerant. (91, GA) (10 ml juice in 250 ml water + Mishri (Sugar lumps)*,* 10 g) Whole plant extract / juice useful in fever and diabetes. (1, FI) Leaves paste applied in cuts and wounds. (1, DE) Stem is used to cure diabetes (5–10 cm stem piece chewed daily). (35, DI)	128	1▲,2▲,3●,4●,5∆,6●,7●,8∆,9●,10●,11●,12●,13●,14∆,15●,16●,17●,18∆,19∆,20●,21●,22∆,23●,24●,25●,26●,27▲,28●,29●,30▲,31●,32●,33●,34●,35●
Musaceae						
*Musa balbisiana* Colla ASR HAPPRC 1614	Kaila/Banana	T	Bark, Fruit	Bark extract (juice) / *rasa* is used as refrigerant (cooling effect). (13, GA) Immature fruit is also used for treatment of dysentery and diarrhea. (11, GA)	24	1●,2∆,3●,4●,5●,6●,7●,8∆,9●,10●,11●,12●,13●,14●,15●,16●,17●,18▲,19●,20●,21●,22●,23●,24●,25●,26●,27∆,28●,29●,30●,31●,32●,33●,34●,35●
Myricaceae						
*Myrica esculenta* Buch.-Ham. ex D. Don ASR HAPPRC 1476	Kaafal/Box myrtle	T	Bark, Root	Bark powder (*churna*) ½-1 teaspoon is taken with lukewarm water thrice a day for treatment of stomachache. (9, GA) Bark extract / juice used to cure cuts and wounds. (17, DE) Root paste (*lepa*) applied for treatment of headache. (6, HA)	32	1●,2∆,3▲,4●,5●,6●,7●,8●,9▲,10●,11●,12●,13●,14●,15▲,16●,17●,18▲,19●,20●,21●,22●,23∆,24●,25●,26●,27∆,28∆,●29●,30●,31●,32●,33●,34●,35●
Myrtaceae						
*Psidium guajava* L. ASR HAPPRC 1610	Amrood/Guava	T	Leaves	Leaves (2–3) rubbed with water, mixed in 250 ml water, and prepared extract is taken orally twice a day to cure stomachache. (21, GA) Leaves’ semi-dried paste (*avleha*) 2–3 teaspoon taken thrice a day with 250 ml water for treatment of dysentery and diarrhea. (22, GA)	43	1●,2●,3▲,4●,5●,6●,7●,8●,9●,10●,11●,12●,13●,14∆,15●,16●,17●,18∆,19●,20∆,21●,22●,23●,24●,25●,26●,27▲,28●,29●,30∆,31●,32●,33●,34▲,35●
*Syzygium cumini* (L.) Skeels ASR HAPPRC 1597	Jaamun/Java Plum	T	Bark, Root	Jaamun bark crushed with water, filtered through cloth and 10 ml (2 tablespoon) taken with 250 ml water thrice a day for treatment of dysentery and diarrhea. (14, GA) Root and bark paste applied for treatment of headache. (11, HA)	25	1●,2●,3▲,4●,5●,6∆,7●,8∆,9●,10●,11●,12●,13●,14∆,15●,16●,17∆,18●,19●,20●,21●,22●,23∆,24●,25●,26●,27▲,28●,29●,30▲,31●,32●,33●,34●,35▲
Orchidaceae						
*Dactylorhiza hatagirea* (D. Don) Soo ASR HAPPRC 1621	Hathajadi/Himalayan Marsh Orchid	H	Tuber, Leaves	Tuber paste (*lepa*) applied on cut and wounds as antiseptic. (14, DE) Leaves rubbed and ½ teaspoon semi-dried preparation (*avleha*) taken orally with 1 glass water for treatment of abdominal heat or as refrigerant. (20, GA) Tuber powder ½-1 teaspoon taken with milk or water to act as tonic. (39, DU)	73	1●,2●,3▲,4●,5●,6▲,7●,8●,9▲,10▲,11●,12▲,13●,14▲,15●,16●,17●,18●,19●,20●,21●,22∆,23▲,24●,25●,26●,27●,28●,29●,30▲,31●,32▲,33▲,34●,35∆
Oxalidaceae						
*Oxalis corniculata* L. ASR HAPPRC 1490	Bhilmod/creeping woodsorrel	H	Aerial part	Aerial parts crushed with lukewarm water, filtered through cloth and 1–2 drops of the fresh juice (*swarasa*) are used to cure earache. (14, EC) Areal part paste (*lepa*) is used for treatment of pimples, skin diseases, cuts and wounds, burns (11, DE). Aerial parts juice (*swarasa*) is used to cure cataract (*ankh me phool*). (9, OP) Aerial parts or stem pieces used to cure boils. (12, DE)	46	1∆,2∆,3▲,4●,5∆,6▲,7●,8●,9∆,10●,11▲,12●,13●,14●,15●,16●,17▲,18●,19●,20●,21▲,22●,23▲,24▲,25●,26∆,27▲,28▲,29●,30▲,31∆,32●,33●,34●,35●
Paeoniaceae						
*Paeonia emodi* Royle ASR HAPPRC 1613	Dhandroo / Gandhya/Himalayan Peony	H	Leaves	1 teaspoon leaves decoction given thrice a day for treatment of child stomachache (*jonku*) (12, GA) and vermifuge (expelling or destroying intestinal worms). (17, GA) It is also used to cure fever. (20, FI)	49	1●,2▲,3∆,4●,5●,6▲,7●,8●,9∆,10●,11●,12●,13●,14●,15∆,16●,17●,18●,19●,20●,21●,22∆,23●,24●,25∆,26●,27∆,28●,29●,30∆,31●,32●,33●,34●,35●
Phyllanthaceae						
*Phyllanthus emblica* L. ASR HAPPRC 1400	Aanwla/Indian gooseberry	T	Fruit	Crushed 3–4 fruits and soaked in water (250 ml) for 1 night then filtered through cloth and the prepared extract / juice (*rasa*) taken orally once a day, acting as refrigerant (cooling effect). (51, GA)	51	1∆,2∆,3●,4●,5∆,6∆,7●,8●,9●,10●,11∆,12●,13●,14∆,15●,16●,17●,18∆,19●,20●,21●,22●,23∆,24▲,25∆,26●,27∆,28●,29●,30▲,31●,32●,33●,34●,35●
Pinaceae						
*Cedrus deodara* (Roxb. ex D. Don) G. Don ASR HAPPRC 1574	Devdaar/Himalayan cedar	T	Bark, Resin	Bark powder (*churna*) ½-1 teaspoon with lukewarm water taken orally thrice a day for treatment of abdominal problem. (11, GA) Leaf and resin paste applied in boils, cuts and wounds. (7, DE) Resin applied for treatment of cracked feet. (6, DE)	24	1●,2●,3●,4●,5●,6●,7●,8●,9●,10●,11●,12▲,13●,14∆,15●,16●,17●,18∆,19●,20●,21●,22∆,23▲,24●,25●,26●,27∆,28●,29●,30∆,31●,32∆,33∆,34●,35∆
*Pinus roxburghii* Sarg. ASR HAPPRC 1580	Cheed / Kulain/longleaf Indian pine	T	Root, Resin	2–3 year old plant root (2–4 g) extract / juice with a dose of 1–2 teaspoon taken orally thrice a day for treatment of tuberculosis. (1, RE) Resin is used for cracked feet, cuts and wounds, and bone fracture. (41,27, DE, SK)	69	1∆,2▲,3●,4●,5●,6●,7●,8●,9●,10●,11●,1213●,14●,15▲,16∆,17●,18∆,19●,20●,21●,22●,23▲,24●,25●,26∆,27∆,28●,29●,30●,31●,32▲,33●,34●,35∆
Plantaginaceae						
*Picrorhiza kurroa* Royle ex Benth. Syn *Neopicrorhiza scrophulariiflora* (Pennell) D.Y.Hong ASR HAPPRC 1432	Kadway/Picrorrhiza	H	Root, Leaves	Root or stolon paste (*lepa*) applied in cuts and wounds, boils, burns and burning sensation, headache (leaves paste also used for same action). (7,15,13 DE,HA) Fresh or dried root extract / juice (*swarasa*) 1 teaspoon taken orally thrice a day for treatment of fever (81 FI), and also used as refrigerant. (42, GA) Root dipped in cow urine (2–4 h) and used for treatment of pimples. (6, DE) ½-1 tablespoon root powder taken once a day early morning before eating to remove intestinal worms. (11, GA) Root extract / juice (*swarasa*) is also beneficial for milk feeding mother. (3, GY) Root extract / juice 1 teaspoon taken orally with lukewarm water for treatment of stomachache. (42, GA)	220	1●,2●,3▲,4●,5∆,6▲,7●,8●,9▲,10▲,11●,12▲,13●,14▲,15▲,16●,17●,18●,19●,20●21●,22▲,23▲,24●,25●,26●,27●,28●,29●,30▲,31●,32▲,33●,34●,35●
*Plantago depressa* Willd. ASR HAPPRC 1468	Syamatu	H	Whole plant	Leaves paste applied for treatment of herpes, and burns. (2, DE) Root paste (*lepa*) and extract / juice (*swarasa*) applied for treatment of boils, and skin diseases. (22,5, DE) Semi-solid preparation (*avleha*) of seeds (seeds crushed with ghee (clarified butter)) ½-1 teaspoon is taken orally thrice a day with lukewarm water for curing indigestion, constipation. (6,2, GA)	37	1●,2▲,3●,4●,5●,6●,7●,8●,9●,10●,11●,12●,13●,14●,15●,16●,17●,18●,19●,20●,21●,22▲,23●,24●,25●,26●,27∆,28●,29●,30●,31●,32●,33●,34●,35●
Poaceae						
*Cynodon dactylon* (L.) Pers. ASR HAPPRC 1625	Dublu / Doob/Bermuda Grass	H	Whole plant	Root rubbed and dipped in water for 4–5 h then ½-1 glass drunk thrice a day for refrigerant quality. (17, GA) Aerial part paste (*lepa*) applied in treatment of headache, cuts and wounds, and skin disease. (26, DE)	43	1●,2∆,3∆,4∆,5∆,6●,7∆,8●,9●,10●,11∆,12∆,13●,14●,15●,16●,17●,18∆,19∆,20▲,21●,22●,23▲,24∆,25●,26●,27∆,28∆,29▲,30●,31●,32●,33●,34●,35∆
*Echinochloa frumentacea* Link ASR HAPPRC 1589	Jhangora/Indian barnyard millet	H	Seeds, Stem	*Bhaat* (cooked like rice) made by *jhangora* seeds is used to cure jaundice. Sometimes it is given with whey or butter milk for similar effect. (79, GA)	79	1●,2●,3●,4●,5●6●,7●,8●,9●,10●,11●,12▲,13●,14●,15●,16●,17●,18●,19●,20●,21●,22●,23●,24●,25●,26●,27∆,28●,29●,30▲,31●,32●,33●,34●,35●
*Hordeum vulgare* L. ASR HAPPRC 1405	Jau / Jo/Barley	H	Seeds	Seeds are dipped in water for 6–8 h and then the water is used as refrigerant. (17, GA) Fried seeds’ flour used for remove to weakness (*sattoo*). (9, DU) *Sattva* (solid extract e.g. ash, macerated in water and stayed overnight then strained through cloth and solid matter allowed to settle) prepared through seeds then it is used for treatment of stomachache, indigestion. (3, GA)	29	1●,2∆,3●,4●,5●6●,7●,8●,9●,10●,11●,12●,13●,14●,15●,16●,17●,18∆,19●,20●,21●,22●,23●,24●,25●,26●,27∆,28●,29●,30●,31●,32●,33●,34●,35●
Polygonaceae						
*Polygonum capitatum* Buch.-Ham. ex D.Don Syn- *Persicaria capitata* (Buch.-Ham. ex D. Don) H. Gross ASR HAPPRC 1568	Lohchadi/pinkhead smartweed	H	Aerial part	Leaves rubbed with mustard oil and the prepared paste is applied in the treatment of herpes. (1, DE) Aerial part paste (*lepa*) applied for treatment of boils and burns. (21, DE)	22	1●,2●,3●,4●,5●6●,7●,8●,9●,10●,11●,12●,13●,14●,15●,16●,17●,18●,19●,20●,21●,22●,23●,24●,25●,26●,27●,28●,29●,30∆,31●,32●,33●,34●,35●
*Rheum emodi* Wall. ex Meisn. Syn. *Rheum australe* D. Don ASR HAPPRC 1549	Archu/Rhubarb	H	Root, Leaves	Fresh or dried root extract / juice 10 ml with 250 ml water taken twice a day as refrigerant. (41, GA) Root powder ½-1 teaspoon taken with water for treatment of internal body injury. (31, DU) Fresh root and leaves paste applied for treatment of headache, muscles and boneache, burns, cuts and wounds. (44, HA, SK, DE)	116	1●,2▲,3▲,4●,5●6●,7●,8●,9▲,10▲,11●,12●,13●,14▲,15●,16●,17●,18●,19●,20●,21●,22▲,23∆,24●,25∆,26●,27●,28●,29▲,30●,31●,32▲,33∆,34●,35▲
*Rumex hastatus* D. Don ASR HAPPRC 1522	Amedu/Arrowleaf Dock	H	Whole plant	Shade dried root powder (*churna*) ½-1 teaspoon taken orally thrice a day for treatment of stomachache. (21, GA) Aerial parts extract / juice used for treatment of burns, cuts and wounds. (18, DE)	39	1●,2∆,3∆,4●,5●6▲,7●,8●,9●,10●,11▲,12●,13●,14●,15●,16▲,17●,18●,19●,20●,21●,22●,23●,24●,25∆,26▲,27▲,28∆,29●,30●,31●,32●,33∆,34●,35∆
*Rumex nepalensis* Spreng. ASR HAPPRC 1603	Khuldya/Nepal Dock	H	Root, Leaves	Leaf and root paste applied in burns, cuts and wounds, skin diseases and boils. (5,9,6,12, DE) Root powder ½-1 teaspoon is taken orally thrice a day for treatment of body pain. (2, DU) Root paste applied for treatment of toothache. (1, DP) *Sattva* (solid extract e.g. root powder (5–10 g), macerated in water (250 ml), stayed overnight, and then strained through cloth and solid matter allowed settle) filtered water (250 ml) drunk once a day as refrigerant and solid matter / powder ½-1 teaspoon taken with water for treatment of stomachache and fever. (5,3, GA, FI)	43	1●,2●,3●,4●,5●6▲,7●,8●,9∆,10●,11●,12●,13●,14●,15∆,16●,17●,18●,19∆,20●,21●,22∆,23▲,24●,25●,26●,27∆,28●,29●,30▲,31●,32●,33∆,34●,35●
Ranunculaceae						
*Aconitum balfourii* Stapf Syn- *Aconitum lethale* Griff. ASR HAPPRC 1424	Bikh	H	Tuber	Tuber paste with *ghee* (clarified butter) applied for treatment of snake bite and scorpion sting, boils, gout, joint pain and body pain (*sool*). Fresh or dried tuber extract / juice also used for same action. (62, 7,3, PB, DE,SK)	72	1●,2●,3●,4●,5●6∆,7●,8●,9∆,10∆,11●,12∆,13●,14●,15●,16●,17●,18●,19●,20●,21●,22▲,23▲,24●,25∆,26●,27∆,28●,29●,30●,31●,32∆,33●,34●,35▲
*Aconitum heterophyllum* Wall. ex Royle ASR HAPPRC 1426	Atees/Indian Atees	H	Tuber	Tuber paste applied for treatment of cut and wounds, boils, headache. (25, DE, HA) Fresh or dried tuber extract / juice dosage of 1 teaspoon taken orally with lukewarm water thrice a day for treatment of fever, stomach ache, and killing intestinal worms. (78, 7, FI, GA) Tuber rubbed with milk and honey, prepared semi-dried (*avleha*), used to cure child fever, stomachache etc. (37,3 FI, GA) Dried or fresh tuber extract or juice dose of ½-1 spoon taken orally thrice a day with lukewarm water taken before meal to cure dysentery and diarrhea. (3, GA)	153	1●,2▲,3▲,4●5●6▲,7●,8●,9▲,10▲,11●,12▲,13●,14▲,15▲,16●,17●,18●,19●,20●21●,22▲,23▲,24●,25●,26●,27●,28●,29▲,30▲,31▲,32▲,33●,34●,35●
*Delphinium denudatum* Wall. ex Hook. f. & Thomson ASR HAPPRC 1417	Nirbishi	H	Root	Root paste (*lepa*) applied for treatment of boils, pimples, cuts and wounds. (22,3,3, DE) Root paste with *ghee* (clarified butter) applied for treatment of scorpion and snake bite. (18, PB)	46	1●,2●,3∆,4●,5●6▲,7∆,8●,9●,10●,11●,12▲,13●,14●,15●,16●,17●,18▲,19●,20●,21●,22●,23∆,24●,25∆,26●,27▲,28●,29●,30▲,31●,32∆,33●,34●,35●
*Thalictrum foliolosum* DC. ASR HAPPRC 1562	Mamiri / Peelijad/Leafy Meadow-Rue	H	Whole plant	Leaf and root extract / swarasa (fresh juice) or paste applied for treatment of boils, skin diseases, cuts and wounds. It also heals burns. (42,4,8, DE)	54	1●,2●,3●,4●,5●,6●,7●,8●,9∆,10●,11●,12●,13●,14●,15●,16●,17●,18▲,19●,20●,21●,22●,23∆,24●,25●,26∆,27∆,28∆,29●,30●,31●,32●,33●,34●,35●
Rosaceae						
*Duchesnea indica* (Jacks.) Focke ASR HAPPRC 1575	Bhuikafal/Indian Strawberry	H	Fruit	Fruit paste (*lepa*) applied for treatment of white patches, and skin diseases. (12, DE) 4–5 fruits rubbed and mix with water (250 ml) taken once a day, as it acts as refrigerant (cooling effect). (14, GA)	26	1●,2●,3●,4●,5●6●,7●,8●,9●,10●,11●,12●,13●,14●,15●,16●,17∆,18●,19●,20●,21●,22●,23●,24●,25●,26∆,27∆,28∆,29●,30●,31●,32●,33●,34●,35●
*Potentilla fulgens* Wall. ex Sims Syn *Potentilla lineata*Trevir. ASR HAPPRC 1553	Bajradanti/ Silver weed	H	Whole plant	Roots and leaves used for cleaning teeth and also used for treatment of toothache. (79, DP) Leaves are chewed to cure throat infection (*khod*). (15, RE)	94	1●,2▲,3▲,4●,5●6●,7●,8●,9▲,10●,11●,12▲,13●,14●,15●,16∆,17●,18●,19●,20●,21●,22∆,23▲,24●,25●,26●,27∆,28●,29●,30●,31●,32▲,33●,34●,35▲
*Prunus persica* (L.) Batsch ASR HAPPRC 1437	Aaru/Peach	T	Bark, Leaves, Seeds	Seeds with pericarp rubbed in *chonthri*, prepared paste is applied in boils and skin diseases. (12, DE) Fine seed (1) powder gently mix in 20 ml water, filter it through cloth then 1 tablespoon given for child as refrigerant (cooling effect). (30, GA)	42	1●,2●,3●,4●,5●6●,7●,8●,9●,10●,11●,12●,13●,14●,15●,16●,17●,18∆,19●,20●,21∆,22●,23●,24●,25●,26●,27∆,28●,29●,30∆,31●,32●,33●,34●,35●
*Rubus ellipticus* Sm. ASR HAPPRC 1444	Hisaur/Golden Himalayan raspberry	S	Root, Leaves, Fruit	Young shoots are chewed for treatment of throat infection (*khod*). (17, RE) Root and leaves paste applied for treatment of skin diseases, and boils. (9, DE) Stem is used as toothbrush for cleaning teeth. (26, DP)	52	1●,2∆,3●,4●,5●6●,7●,8●,9●,10●,11●,12●,13●,14●,15●,16●,17∆,18●,19●,20●,21●,22●,23∆,24●,25●,26∆,27∆,28∆,29●,30●●,31●,32●,33●,34●,35●
Rubiaceae						
*Rubia manjith* Roxb. ex FlemingASR HAPPRC 1473	Lyachkuru/Indian madder	Cl	Whole plant	Aerial plant paste applied for treatment of skin diseases, burns, boils and headache. (7,6,3, DE, HA) Whole plant powder (*churna*) ½-1 teaspoon with lukewarm water is taken thrice a day for treatment of abdominal problems. (3, GA)	19	1●,2●,3●,4●,5∆,6●,7●,8●,9∆,10●,11●,12●,13●,14●,15∆,16∆,17●,18∆,19∆,20●,21●,22▲,23▲,24●,25●,26●,27∆,28∆,29●,30●,31●,32●,33●,34●,35●
Rutaceae						
*Citrus aurantiifolia* (Christm.) Swingle ASR HAPPRC 1579	Kaagji/Lime	S	Fruit	1 Fruit juice prepared with 250–500 ml water + ½-1 teaspoon salt +5–10 g Mishri (Sugar lumps) (sugar lumps) taken orally for treatment of dysentery and diarrhea, acts as a refrigerant (cooling effect) (42, GA), and it is also used to cure fever and headache. (29, FI, HA) Fruit juice applied for treatment of pimples, cuts and wounds. (9, DE)	80	1●,2●,3●,4●,5●,6●,7●,8●,9●,10●,11●,12●,13●,14●,15●,16●,17●,18●,19●,20●,21●,22●,23●,24●,25●,26●,27∆,28●,29●,30●,31●,32●,33●,34●,35●
Saxifragaceae						
*Bergenia ciliata* (Haw.) Sternb. ASR HAPPRC 1578	Pashanbhed / Syalmadi / Kaamal/Frilly Bergenia	H	Root, Leaves	Fresh (5 g) or dried (2 g) root ground with *ghee* (clarified butter) (1 teaspoon) mixed with 250 ml water, taken once a day for abdominal sanitation. (3, GA) Root and leaf paste is used for treatment of burns, boils, cuts and wounds. (7, DE) Root juice (*swarasa*) 1 teaspoon in 250 ml water used as refrigerant (cooling effect). Root ground with water, made into semi dried preparation, then ½ teaspoon is given with milk to child thrice a day to cure *syalbey* (when child go to cool side rapidly or kind of fever). Root decoction also used for cure stone (8, FI, GA) Root is also useful in leucorrhoea. (4, GY) Root powder (½-1 teaspoon) taken thrice with lukewarm water for cure stomachache and stone (*pathri*). (45, GA)	67	1●,2▲,3●,4●,5●,6∆,7●,8●,9∆,10▲,11●,12▲,13●,14●,15▲,16▲,17●,18▲,19●,20●,21●,22∆,23▲,24●,25∆,26●,27▲,28▲,29●,30▲,31▲,32●,33∆,34●,35●
Smilacaceae						
*Smilax aspera* L. ASR HAPPRC 1448	Kukrdaad/Common smilax	Cl	Fruit	Fruit (7–9) + 1 tablespoon Ghee (clarified butter) paste (*lepa*) applied for treatment of snake bite and scorpion sting for 5 days. (2, PB)	2	1●,2●,3●,4●,5●,6●,7●,8●,9●,10●,11●,12●,13●,14●,15●,16●,17●,18∆,19●,20●,21●,22●,23●,24●,25●,26∆,27∆,28∆,29●,30●,31●,32●,33●,34●,35●
Solanaceae						
*Solanum khasianum* C.B. Clarke Syn- *Solanum aculeatissimum* Jacq. ASR HAPPRC 1583	Bhugundroo / Konldbey/Dutch eggplant	S	Fruit, Root	Fruit garland is used to cure jaundice. (61, GA) Root decoction (½-1 teaspoon) taken thrice a day for 5–7 days to cure jaundice (*konlbey*). (1, GA) Root paste applied to cure boils and burns. (14, DE)	76	1●,2▲,3●,4●,5●,6●,7●,8●,9●,10●,11●,12●,13●,14●,15●,16●,17●,18●,19●,20●,21●,22●,23●,24●,25●,26●,27▲,28●,29●,30●,31●,32●,33●,34●,35●
*Solanum nigrum* L. Syn- *Solanum americanum* Mill ASR HAPPRC 1459	Kiwaini / Kyawen/Black nightshade	H	Fruit, Leaves	Mature fruit (4–5) juice (*swarasa*) mixed with 250 ml water taken orally twice a day to cure fever, indigestion, and acts as refrigerant (cooling effect). (11,9, 16, FI, GA) Fruit paste (*lepa*) applied on forehead for treatment of headache. (1, HA) Leaves juice (*swarasa*) applied in cuts and wounds, boils. (2, DE)	39	1▲,2▲,3●,4●,5∆,6●,7●,8●,9●,10●,11●,12●,13●,14●,15●,16●,17∆,18▲,19▲,20▲,21●,22●,23●,24▲,25●,26●,27▲,28∆,29●,30▲,31▲,32●,33∆,34●,35●
Taxaceae						
*Taxus wallichiana* Zucc. ASR HAPPRC 1607	Thuner/Himalayan yew	T	Leaves	Leaves extract / juice applied for treatment of boils, cuts and wounds. (27,15, DE)	42	1●,2●,3●,4●,5●,6∆,7●,8●,9∆,10●,11●,12∆,13●,14●,15∆,16●,17∆,18●,19●,20●,21●,22∆,23∆,24●,25●,26●,27∆,28●,29●,30∆,31●,32●,33∆,34●,35●
Urticaceae						
*Girardinia diversifolia* (Link) Friis ASR HAPPRC 1618	Dholan/Himalayan nettle	H	Whole plant	Root decoction is used for treatment of boils, swelling and joint pain. (10, 9,4, DE, SK) Fresh root is also used for treatment of boils. (6, DE)	29	1●,2∆,3●,4●,5●,6●,7●,8●,9∆,10∆,11●,12●,13●,14●,15●,16●,17●,18●,19●,20●,21●,22●,23∆,24●,25●,26●,27∆,28●,29●,30▲,31●,32●,33▲,34●,35●
*Pouzolzia hirta* Blume ex Hassk. ASR HAPPRC 1628	Kanchwalya	H	Root	Root paste used to remove dandruff and prevent hair fall. (92, HP)	92	1●,2●,3●,4●,5●,6●,7●,8●,9●,10●,11●,12●,13●,14●,15●,16●,17●,18●,19●,20●,21●,22●,23●,24●,25●,26●,27●,28●,29●,30●,31●,32●,33●,34●,35●
Violaceae						
*Viola canescens* Wall. ASR HAPPRC 1537	Bansai/Banasa/Himalayan White Violet	H	Aerial part	Aerial plant paste used for cuts and wounds, (9, DE), flowers powder (*churna*) ½-1 teaspoon taken orally thrice a day with lukewarm water to cure cough. (11, RE)	20	1●,2●,3●,4●,5●,6●,7●,8●,9▲,10●,11●,12●,13●,14●,15▲,16●,17●,18●,19●,20●,21●,22●,23●,24●,25●,26▲,27∆,28▲,29●,30▲,31∆,32●,33●,34●,35●
Zingiberiaceae						
*Curcuma longa* L. ASR HAPPRC 1619	Haldu/Turmeric	H	Rhizome	Rhizome paste applied in cuts and wounds acts as antiseptic. (87, DE) To cure deep bone wounds and internal body injury rhizome powder ½ teaspoon (1 g) mixed with 1 glass milk is drunk 1 glass a day. (19, DU)	106	1●,2●,3●,4●,5●,6●,7●,8●,9●,10●,11●,12▲,13●,14●,15●,16●,17●,18∆,19●,20▲,21●,22●,23●,24▲,25●,26●,27▲,28●,29●,30∆,31●,32●,33●,34∆,35●
*Hedychium spicatum* Sm. ASR HAPPRC 1416	Syodu / Banhaldu/Spiked Ginger Lily	H	Rhizome, leaves	Rhizomes (40-50 g) boiled in 100 ml water then the paste is applied for treatment of joint pain, burns, boils, and skin diseases. (4, SK, DE) Fresh rhizome extract / juice can be used for treatment of cuts and wounds and boils. (22, DE) Leaves paste (*lepa*) applied for treatment of headache. (6, HA)	32	1●,2∆,3∆,4●,5●,6●,7●,8●,9∆,10●,11●,12●,13●,14●,15●,16∆,17●,18∆,19∆,20●,21●,22∆,23∆,24●,25∆,26∆,27∆,28∆,29●,30∆,31●,32●,33●,34●,35●
*Zingiber officinale* Roscoe ASR HAPPRC 1609	Aadu/Ginger	H	Rhizome	Rhizome powder (½-1) teaspoon taken orally thrice a day with lukewarm water for treatment of cough and cold. (66, RE) Rhizome paste (*lepa*) also used for curing burns and boils. (6,2, DE)	74	1∆,2●,3▲,4●,5∆,6●,7●,8●,9●,10●,11●,12∆,13●,14∆,15●,16●,17●,18∆,19●,20●,21●,22●,23●,24●,25∆,26●,27∆,28●,29●,30▲,31●,32●,33●,34▲,35●

*LF* life forms, *H* herb, *S*, shrub; *T* tree, *Cl* climber

*GA* gastro-intestinal disorders,*RE* respiratory complaints, *FI* fever and aches, *DE* Diseases of the skin, *GY* women’s health, *SK* skeletomuscular disorders, *DI* diabetes, *OP* ophthalmologic complaints, *PB* poisonous bite, *DP* dental problems, *HP* Hair problems, *EC* ear complaints, *HA* head ache, *MA* mental afflictions, *DU* different uses

(▲) Similar use, (Δ) Dissimilar use, and (●) Not reported

1 [35]2 [31]3 [30]4 [41]5 [43] 6 [1],7 [48]8 [46]9 [22] 10 [26], 11 [49] 12 [32], 13 [50] 14 [51]15 [52], 16 [53]17 [54], 18 [39]19 [55] 20 [56]21 [57] 22 [38]23 [45], 24 [33]25 [58] 26 [29] 27 [11], 28 [27] 29 [59], 30 [44], 31 [28], 32 [36], 33 [37], 34 [17], 35 [18]

### Life forms and plant parts used

**Fig. 4:**
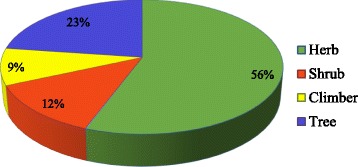
Proportion of different life forms used as medicinal plants in Jakholi

In present study, 56% of the species were herbs, followed by trees (23%), shrubs (12%), and climbers (9%) (Fig. 4), similar to other studies carried out in Himalaya [1, 17, 18], probably due to the presence of a wide range of rich bioactive medicinal plants in the Himalaya [19]. Traditional healers often use herbs and trees most commonly as medicine because of their easy availability [20]. Besides this, herbs can be manipulated with easiness in herbal preparation methods and extraction of bioactive compounds [21]. Less percentage of climbers might be due to less availability and difficult to harvest from huge growth of supporting material (Tree) in temperate area. Availability is found as a major reason to use the plants in Himalaya followed by cultural reason.

**Fig. 5:**
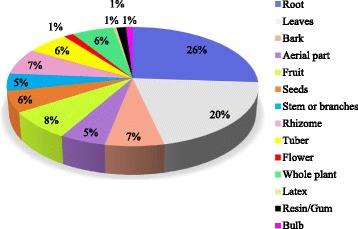
Proportion of different plant parts used for ethnomedicinal purpose in Jakholi

In present study different plant parts were used to prepare herbal preparation of drugs (Fig. 5). The common plants parts were roots (26%) followed by leaves (20%), fruit (8%), bark and rhizome (7%) whole plant, tuber and seeds (each 6%), aerial part and stem or branches (each 5%), flower, latex resin or gum, bulb, (each 1%). Root were frequently used in folklore of Jakholi for herbal preparations similar to [1, 22] Root proportion is high probably due to root consist rich of active ingredients [23]. Leaves were second most useful plant part it might be due to easy availability and it is thought that leaves contain more easily extractable phytochemicals, crude drugs and many other mixtures which may be proven as valuable regarding phytotherapy [24].

### Mode of drug preparation and traditional tools

**Fig. 6:**
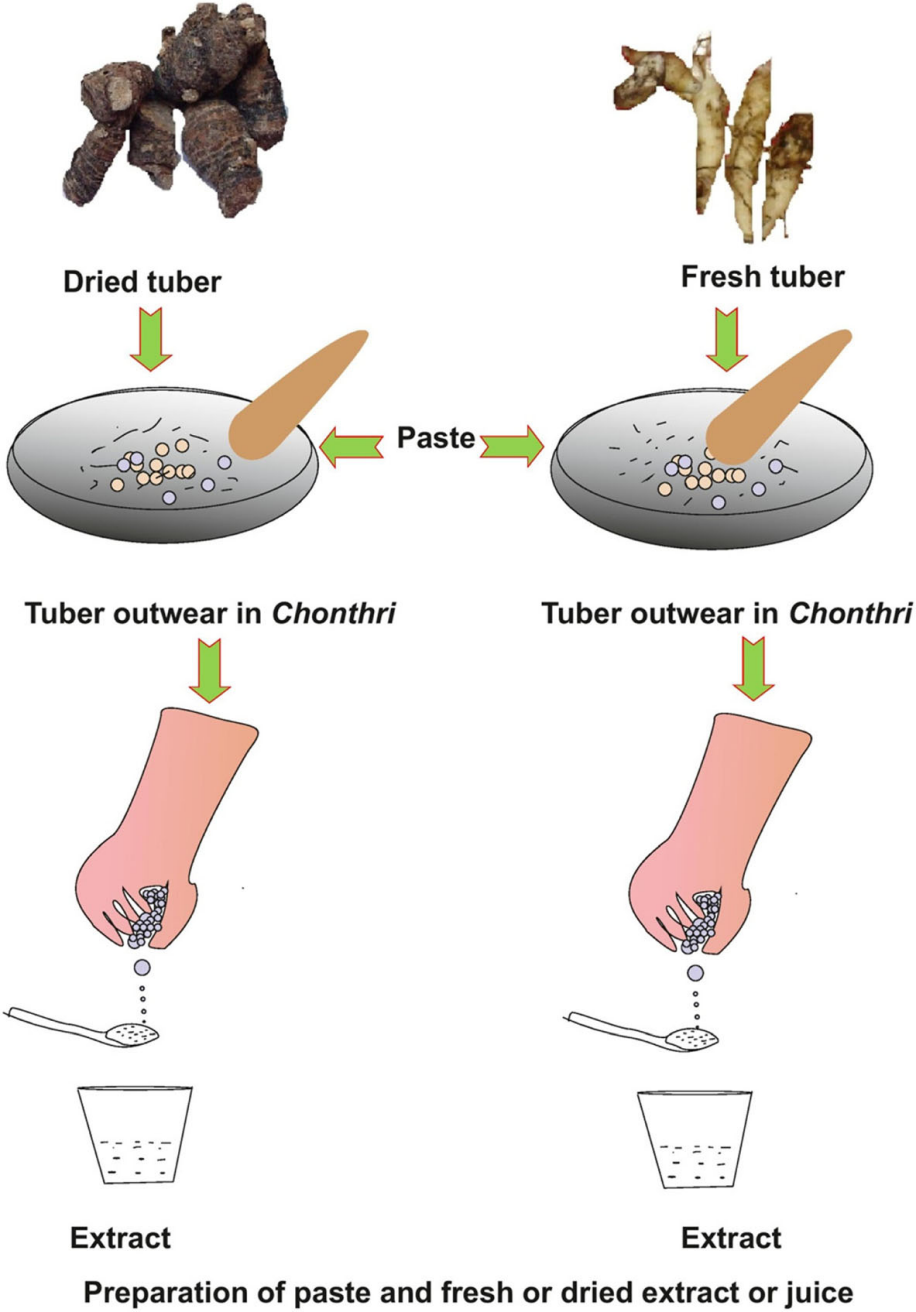
Paste (Lepa) and Extract (Rasa) preparation by local inhabitants of Jakholi

**Fig. 7:**
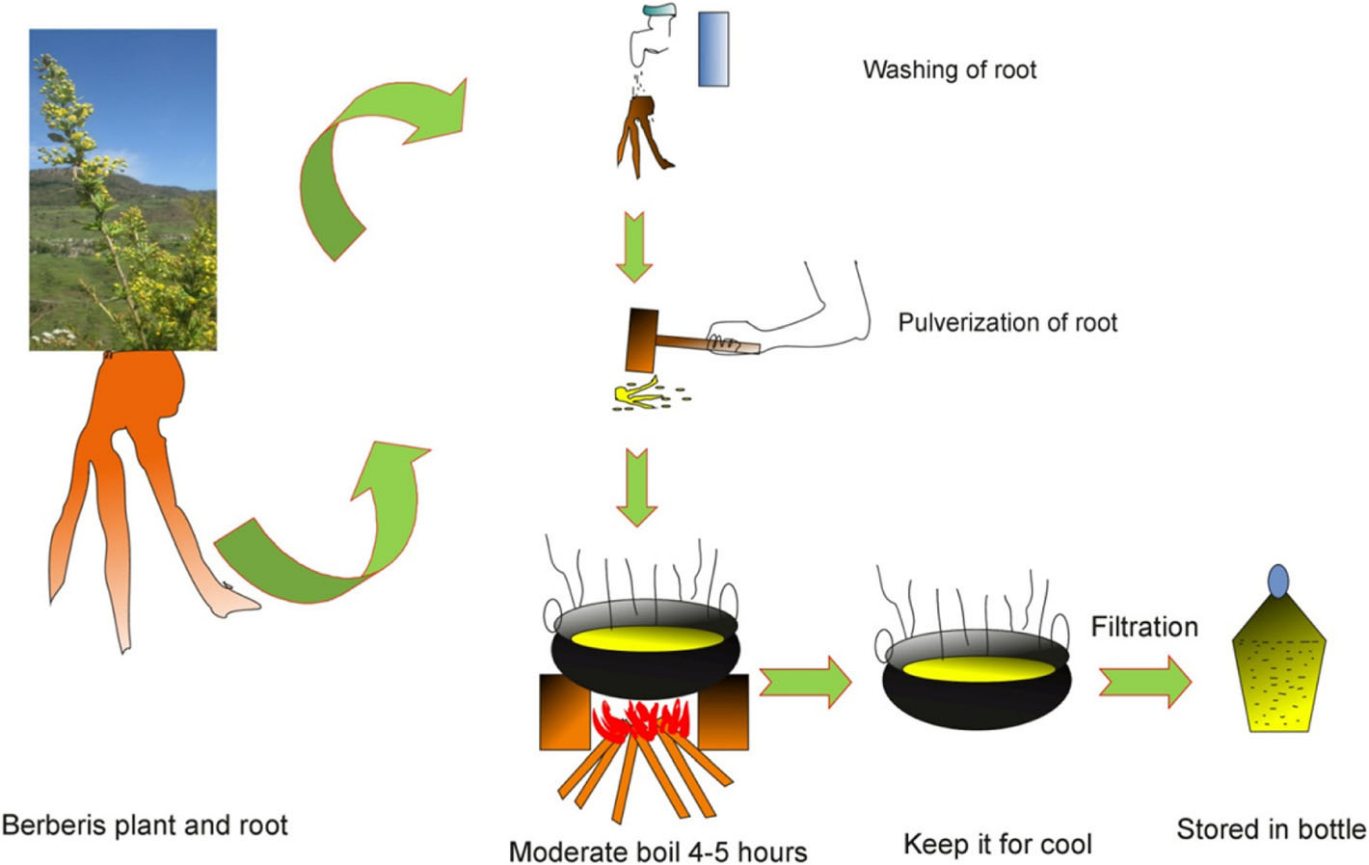
Decoction (Rasout) preparation by local inhabitants of Jakholi

**Fig. 8:**
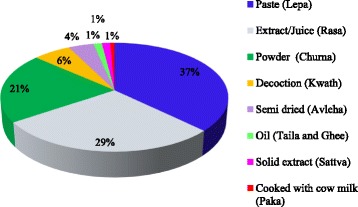
Proportion of different formulations of medicinal plants in Jakholi

Out of total 148 preparations, the herbal medicine formulations prepared according to the traditional uses as follows: paste (lepa) (37%), juice/extract (rasa) (29%), powder (churna, 21%), decoction (kwath/kaada) (6%), semi-dried (avleha) (4%), oil (taila/ghee), solid extract (sattva), and cooked with milk (paka) (each 1%) (Figs. 6, 7 and 8). The most frequent use of paste and juice might be due to easy preparation and effectiveness of herbal drugs. Water was commonly used as solvent if required for the preparation. Sometimes milk or honey was used as a matrix or added to increase viscosity of the preparation as reported in earlier study [25]. Paste is made by crushing plant parts and then mixing it with oil or water. Administration of dosages was taken mostly twice and thrice a day. Besides above, according to few participants the dosage depends on the age and physical appearance of the patient [24].Mostly traditional tools used by local inhabitants for drug preparation are: *Chhonthri* (made of stone in the shape of plate 10 – 12 mm thick and with a diameter of 15 – 20 cm and a weigh of about 0.5 – 1.0 kg (Fig. 2d), *Kharad* (also made of stone 20 cm × 45 cm, 3 – 5 kg weight), *Silbatta/Silotu* (made of stone 30 × 60 cm, 15 – 25 kg weight) (Fig. 2d), *Imaamdasta* (made of stone or readymade china ceramic, 3 – 5 kg weigh).

### Informant consensus factor (ICF)

The consensus of participants on medicinal plants reported for treating different ailments was quantitatively analyzed. To develop this consensus, all treated diseases are grouped into 15categories. ICF value ranged from 0.91 – 0.99, inferring the high consensus value among participants, however the 100% consensus was not reported. The highest ICF value (0.99) was for hair problems (HP), followed by Ophtalmologic complaints (OP) Mental afflictions (MA) 0.98 (Table 4). Our result repudiated the earlier findings and found the highest ICF for HP and OP. It may be due to low availability of market based nutraceuticals and OP was attributed by the poor sanitation, frequent injuries made by scrubs, wind, insects and poisonous flowers/pollens. Low consumption of water, high intensity light, hard work might be one of the important factors causing MA. High ICF values from adjoining areas were recorded for haematological disorder (1.00) [26], Liver disorder (0.56) [27], Malaria, Measles, Giddiness (each 1.00) [28].

**Table 4:** Informant consensus factor for ailment categories

Ailment categories (group of illness)	Number of use reports (Nur)	% of use reports	Number of taxa (Nt)	% of taxa	Informant consensus factor (ICF)
Women’s health (GY)	35	0.70	4	5.12	0.91
Head ache (HA)	199	4.00	14	17.94	0.93
Respiratory complaints (RE)	219	4.40	10	12.82	0.95
Diabetes (DI)	49	0.98	3	3.84	0.95
Diseases of the skin (DE)	1468	29.55	53	67.94	0.96
Skeletomuscular disorders (SK)	128	2.57	6	7.69	0.96
Ear complaints (EC)	104	2.09	5	6.41	0.96
Poisonous bite (PB)	91	1.83	4	5.12	0.96
Gastrointestinal disorders (GA)	1286	25.89	39	50	0.97
Fever and Aches (FI)	437	8.79	15	19.23	0.96
Dental problems (DP)	275	5.53	7	8.97	0.97
Mental afflictions (MA)	71	1.42	2	2.56	0.98
Ophthalmologic complaints (OP)	252	5.07	4	5.12	0.98
Hair problems (HP)	104	2.09	2	2.56	0.99
Different uses (DU)	249	5.01	10	12.82	0.95
TOTAL	4967				

### Ailments and useful species

A total of 4967 therapeutic URs were documented for 15 different ailments categories and the most (1468 reports) were related to diseases of skin (DE) (29.55%). This account was accorded to the findings of Saha et al. [29] confirming that dermatology is the most represented therapeutic category in India, followed by Gastro- intestinal disorder (GA) (25.89%) (Table 4). Women’s health (GY) cited less UR (0.70%).

A total of 1286 URs from 39 medicinal plants were reported to treat gastrointestinal ailments (GA) (killing intestinal worms, dysentery and diarrhoea, refrigerant, stomach ache, abdominal sanitation, indigestion, carminative, and constipation) with ICF value 0.97. *Tinospora cordifolia* was highly cited for refrigerant in this ailments category with 91 URs it is commonly known as Giley. *Echinochloa frumentacea* was frequently cited for jaundice with 79 URs. *Megacarpaea polyandra* used as refrigerant with 56 URs, however Semwal et al. [30] and Singh and Rawat [22] reported it for fever, asthma, stomach ache and dysentery. *Bergenia ciliata* commonly known as Pashanbhed / Syalmadi / Kaamalhighly was cited for curing gallstone with 53 URs, similar account was made by Uniyal and Shiva [31].

Total 219 URs and 10 taxa were cited for respiratory complaints (RE) categories and ICF value is 0.95. Cough and cold, tuberculosis and throat infection use reports were common in RE due to cold, fluctuation in temperature, and high smoking. *Zingiber officinale* commonly known *Aadu*, was highly cited for cough and cold with 66 UR as reported by Semwal et al. [30] for cough and cold with honey. Alien and invasive plant *Eupatorium adenophora* was used for cough and cold with18 URs. A total of 437 URs and 15 taxa were mentioned for fever and aches complaints (FI) categories with ICF value (0.96). *Picrorhiza kurroa* and *Aconitum heterophyllum* highly cited for fever and headaches with 81 and 78 URs, substantiate the findings from Garhwal by Uniyal and Shiva [31], Semwal et al. [30], Malik et al. [1], Singh and Rawat [22]., Highest number of URs (1468) from 53 species for skin diseases (DE) with ICF value (0.96) was noted for treatment of cuts and wounds, boils, burnt, pimples, white patches and herpes. Cut and wounds and boils are commonly occurred in hilly areas due to narrow trails and intensive thorny shrubs, tiresome work with sharp tools and implements, etc. *Eupatorium adenophora was* highly cited for cut and wounds with 108 URs followed by *Curcuma longa* with 87 URs, consistent with the findings of Phondani et al. [32], Tewari et al. [33] and Gaur [11]. Women’s health problems like galactogogue and leucorrhoea were treated by *Asparagus adscendens, Picrorhiza kurroa*, *Bergenia ciliata* and *Quercus leucotrichophora.* This result is consistent with the findings of Azad and Bhat [34]. *Rheum emodii* was highly cited for bone ache with 44 URs as noted by Semwal et al. [30]. *Tinospora cordifolia* was highly cited for diabetes with URs 35 followed by *Berberis chitria* and *Berberis lyceum* with 7 URs for treatment of diabetes. However, Chandra et al. reported *Berberis lyceum* for ophthalmic complaints [35], Uniyal and Shiva for antiseptic, blood purifier, conjunctivitis [31]. Ophthalmologic complaints (OP) was the second highest ICF value recorder. *Berberis chitria* commonly known Totar/Totru root decoction commonly called Rasout 1–2 drops was used to treat eye infection with 110 URs followed by 101 URs of *Berberis lyceum* for eye complaints, similar observations were made in Himalayan areas [1, 28, 36, 37]. *Centella asiatica* was also beneficial for eye sight with 40 URs. The use of plants or poisonous bite (PB) was moderately consented and only 91 URs from 4 taxa were cited for poisonous bite (PB) complaints with ICF value 0.96. *Aconitum balfourii* was used for Snake bite and Scorpion sting with 62 URs as Rana et al. [38] recorded. *Juglans regia* was cited for cleaning teeth and for treatment of pyorrhoea with 89 URs similar to Uniyal and Shiva [31], Semwal et al. [30], Malik et al. [1] Highest consensus was reported for treatment of hair problems. A total of 104 URs from only 2 species *Pouzolzia hirta* and *Brassica juncea* were cited for hair problems. *Pouzolzia hirta* commonly known as *Kanchwalya* tuberous root paste is used as shampoo and highly cited for to remove dandruff and prevent hair fall. *Brassica juncea* was also cited for ear problems with 42 URs similar to Semwal et al. [30] and Kumari et al. [39]. *Rheum emodii* root and leaf paste was cited for headache, consistent with the observation of Rehman et al. [40]. Species *Nardostachys jatamansi* and *Valeriana jatamansi* were cited for mental disorder and insomnia, as evidenced by Semwal et al. [30], Sharma et al. [41] and Shah et al. [29]. In sense of plants used, the highest number was observed for DE categories (67.94%) followed by Gastro- intestinal ailments (GA) (50%). It has been affirmed that the local people are interested to use herbal therapies predominantly for the management of dermatological and gastro-intestinal ailments. The reported plants having high citations against above mentioned diseases should be further evaluated and analyze through pharmaceutical and biological properties [24, 42].

### Threatened species

**Fig. 9:**
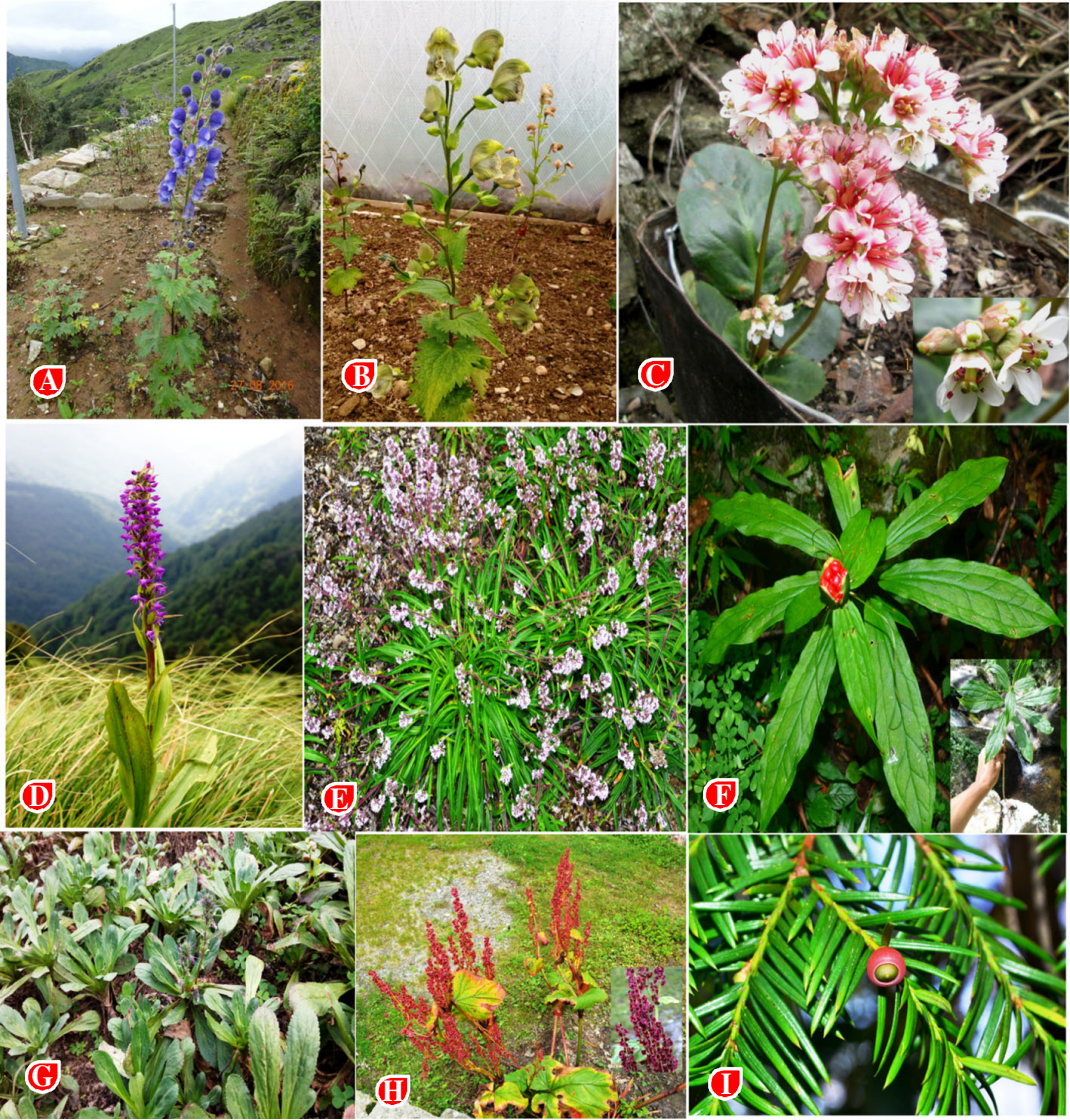
Threatened species in study area **a** *Aconitum balfaourii* **b** *Aconitum heterophyllum* **c** *Bergenia ciliata* **d** *Dactylorhiza hatagirea* **e** *Nardostachys jatamansi* **f** *Paris polyphylla* **g** *Picrorhiza kurroa* **h** *Rheum emodi* **i** *Taxus wallichiana*

**Fig. 10:**
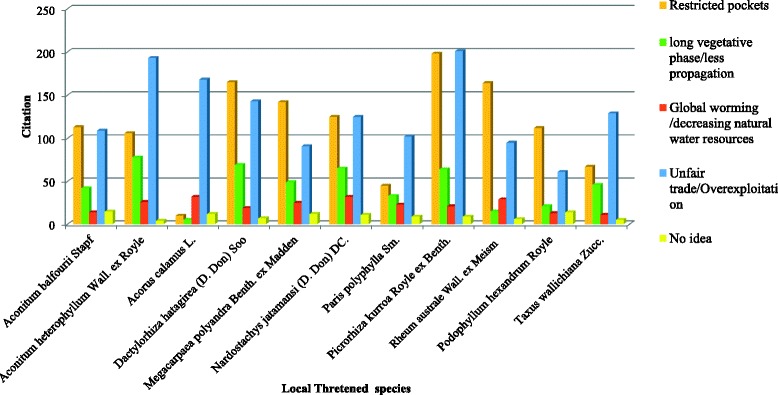
Consensus and observation for local threatened medicinal plants and their causes by local inhabitants of Jakholi

Of the plants recorded for ethnomedicinal, 29 plant species are prioritized for conservation (Table 5). These threatened species are available in restricted pocket of Garhwal Himalaya, and locally threatened due to premature and over-exploitations (Fig. 9). Eleven local highly threatened species were cited by local inhabitants of Jakholi and overexploitation as principle cause of threat cited by local inhabitants for all local threatened species. Alpine species are highly threatened, which may be influence by other cause viz. long vegetative phase and less propagation, decreasing natural water resources and global warming. (Table 6/ Fig. 10)

**Table 5:** Threatened species of Indian Himalayan region used in ethnomedicine practices in study area

S.No	Botanical name	IUCN (1993)[60]	CAMP (Conservation Assessment and Management Plan) (1998) [61]	RDB (Nayar and Shastry, 1987, 1988, 1990) [62]	Gaur (1999)[11]	Dhar et al. (2002) [63]	Nautiyal and Nautiyal (2004) [64]	IUCN (2017)
1	*Aconitum balfourii* Stapf		CR	VU	CR			
2	*Aconitum heterophyllum* Wall. ex Royle	VU	CR			EN	EN	EN
3	*Acorus calamus* L.		VU					LC
4	*Berberis lyceum* Royle		EN					
5	*Berberis chitria* Buch. Hamex Lindl		EN					
6	*Bergenia ciliata* (Haw.) Sternb.		VU		UV			
7	*Betula utilis* D. Don					EN		
8	*Cedrus deodara* (Roxb. ex D. Don) G. Don							LC
9	*Centella asiatica* (L.) Urban							LC
10	*Cinnamomum tamala* (Buch.-Ham.) T. Nees & Eberm.		LR	VU				
11	*Dactylorhiza hatagirea* (D. Don) Soo		CR	EN			R	
12	*Delphinium denudatum* Wall. ex Hook. f. & Thomson		CR					
13	*Engelhardtia spicata* Lechen ex Blume							LC
14	*Girardinia diversifolia* (Link) Friis							
15	*Hedychium spicatum* Sm.		VU					
16	*Juglans regia* L.							NT
17	*Jurinea macrocephala* DC.		LR	VU	R			
18	*Mangifera indica* L.							DD
19	*Megacarpaea polyandra* Benth. ex Madden						VU	
20	*Nardostachys jatamansi* (D. Don) DC.		CR			CR		CR
21	*Paeonia emodi* Royle		VU		VU			
22	*Paris polyphylla* Sm.						VU	
23	*Picrorhiza kurroa* Royle ex Benth.	VU	EN	EN		EN		
24	*Pinus roxburghii* Sarg.							LC
25	*Rheum emodi* Wall. ex Meisn.		VU			VU		
26	*Podophyllum hexandrum* Royle	EN	CR	EN		EN	EN	
27	*Taxus wallichiana* Zucc.		CR			CR		
28	*Thalictrum foliolosum* DC.		VU					
29	*Valeriana jatamansi* Jones		CR	EN				

*CR* critically endangered, *VU* vulnerable, *EN* endangered, *LR* lower risk near threatened, *LC* least concern, *DD* data deficient, *NT* near threatened, R rare

IUCN: The International Union for Conservation of Nature and Natural Resources

http://www.iucnredlist.org 28 May 2017 Data base

**Table 6:** Consensus and observation for local threatened medicinal plants and their causes by local inhabitants of Jakholi

Botanical name	Availability /Citation ∑Citation	Restricted pockets ∑Citation	long vegetative phase/less propagation ∑Citation	Global worming /decreasing natural water resources ∑Citation	Unfair trade/Overexploitation ∑Citation	No idea ∑Citation
*Aconitum balfourii* Stapf	R/98	113	42	14	109	15
*Aconitum heterophyllum* Wall. ex Royle	VR/183	106	78	26	193	4
*Acorus calamus* L.	S/93	10	5	32	168	12
*Dactylorhiza hatagirea* (D. Don) Soo	R/109	165	69	19	143	7
*Megacarpaea polyandra* Benth. ex Madden	R/103	142	49	25	91	12
*Nardostachys jatamansi* (D. Don) DC.	VR/176	125	65	32	125	11
*Paris polyphylla* Sm.	S/91	45	33	23	102	9
*Picrorhiza kurroa* Royle ex Benth.	VR/174	198	64	21	201	9
*Rheum emodi *Wall. ex Meisn. D. Don	R/125	164	15	29	95	6
*Podophyllum hexandrum* Royle	R/81	112	21	13	61	14
*Taxus wallichiana* Zucc.	R/76	67	46	11	129	5

*S* scattered, *R* rare, *VR* very rare (*N* = 220)

.

### Reliability and comparison

Cultural practices of particular region directly affected by floral and faunal wealth and variance among them indicate importance of particular region. For similarity, dissimilarity and new use reports uses of plants documented in our study were compared to 35 published ethno-botanical studies from Indian Himalaya as well as neighbouring countries (Table 7). In the present study, the similarity of uses as compared to other studies ranged from 0 to 30% while dissimilar uses varied widely from 42.5 [43] to 1.58% [44]. JI range between 2.86 – 56.66 and Sorensen’s index 5.56–72.34 were obtain. The highest degree of similarity was found with studies conducted by Kala [45] with JI 55.66 and SI 72.34 and Uniyal and Siva [31] with JI 49.35, SI 66.08. The lowest indices of similarity are found with studies of Samant et al. [36] and Ghildiyal et al. [46] (JI 2.86 and 3, SI 5.56 and 5.83). Comparison of medicinal flora and uses within district and block only two reports were found which have more than 30 JI and 50% SI similarity (49.35 JI, 66.08 SI Uniyal and Siva [31] and 39.68JI, 56.81 SI Semwal et al.) [30]. It appears that the distance between study area and neighbouring region is responsible for any change in JI [24]. The highest similarity index was not surprisingly observed with the nearest areas, which had high similarity indices with respect to plant use and modes of applications.

**Table 7:** Comparison of present study with previous study from adjoining area of Himalaya region

Study area	Study Year	Number of plants reported	Plants with similar use	Plants with dissimilar use	Total Common species in both areas	% of common plants species	Species listed only in aligned areas	Species enlisted only in study area	% of species enlisted only in this study	% of plants with similar uses	% of plants with dissimilar uses	Jaccard index (JI)	Sorensen’s similarity index (QS)	Reference
Rudraprayag district, Uttarakhand	2013	159	7	8	15	9.43	144	63	80.77	4.40	5.03	7.81	14.49	Chandra et al. [35]
Garhwal Himalaya, Uttaranchal	2005	113	24	14	38	33.63	75	40	51.28	21.24	12.39	49.35	66.08	Uniyal and Siva [31]
Ukhimath Block, Rudraprayag Uttarakhand	2010	60	18	7	25	41.67	35	53	67.95	30.00	11.67	39.68	56.81	Semwal et al. [30]
Sub-Himalayan region, Uttarakhand	2013	24	1	7	8	33.33	16	70	89.74	4.17	29.17	10.25	18.6	Sharma et al. [41]
Sub-Himalayan region, Uttarakhand	2012	40	0	17	17	42.50	23	61	78.21	0.00	42.50	25.37	40.47	Sharma et al. [43]
Western Himalaya	2015	97	14	8	22	22.68	75	56	71.79	14.43	8.25	20.18	33.58	Malik et al. [1]
Uttarakhand	2015	56	2	3	5	8.93	51	73	93.59	3.57	5.36	4.2	8.06	Kala [48]
Garhwal region	2014	67	1	3	4	5.97	63	74	94.87	1.49	4.48	3	5.83	Ghildiyal et al. [46]
Kedarnath Wildlife Sanctuary in Western Himalaya, India	2011	126	12	17	29	23.02	97	49	62.82	9.52	13.49	24.78	39.72	Singh and Rawat [22]
Kedarnath Wildlife Sanctuary, India Himalaya	2013	21	6	3	9	42.86	12	69	88.46	28.57	14.29	12.5	22.22	Bhat et al. [26]
Garhwal Himalaya, India	2011	61	8	5	13	21.31	48	65	83.33	13.11	8.20	13	23	Kumar et al. [49]
Niti valley central Himalaya, India	2010	86	9	11	20	23.26	66	58	74.36	10.47	12.79	19.23	32.25	Phondani et al. [32]
Garhwal Himalaya	2010	23	2	1	3	13.04	20	75	96.15	8.70	4.35	3.26	6.31	Dangwal et al. [50]
Uttaranchal, India	2005	74	5	10	15	20.27	59	63	80.77	6.76	13.51	14.01	24.59	Kala et al. [51]
Kedarnath Wildlife Sanctuary, Garhwal Himalaya India	2013	152	11	6	17	11.18	135	61	78.21	7.24	3.95	9.49	17.34	Bhat et al. [52]
Pauri Garhwal Uttarakhand	2010	61	6	6	12	19.67	49	66	84.62	9.84	9.84	11.65	20.86	Pala et al. [53]
Nanital of Kumaun region Uttarakhand	2014	28	3	8	11	39.29	17	67	85.90	10.71	28.57	15.06	26.19	Kapkoti et al. [54]
Almora district Uttarakhand, India	2011	188	10	24	34	18.09	154	44	56.41	5.32	12.77	20.73	34.34	Kumari et al. [39]
Kumaun Himalaya, India	2013	48	3	10	13	27.08	35	65	83.33	6.25	20.83	14.94	26	Bhatt et al. [55]
Bhabar region of Uttarakhand	2015	24	3	4	7	29.17	17	71	91.03	12.50	16.67	8.64	15.9	Pande and Joshi [56]
Sub Himalayan tract Uttarakhand, India	2010	54	2	6	8	14.81	46	70	89.74	3.70	11.11	7.4	13.79	Gaur et al. [57]
Nanda Devi Biosphere reserve, Uttarakhand, India	2013	90	9	16	25	27.78	65	53	67.95	10.00	17.78	26.88	42.37	Rana et al. [38]
Tons watershed Uttarakhand Himalaya	2015	84	17	17	34	40.48	50	44	56.41	20.24	20.24	56.66	72.34	Kala [45]
Garur Block of district Bageshwar, Uttarakhand, India	2014	39	4	4	8	20.51	31	70	89.74	10.26	10.26	8.6	15.84	Tewari et al. [33]
Uttarakhand	2014	111	1	15	16	14.41	95	62	79.49	0.90	13.51	11.34	20.38	Prakash [58]
Nanital Uttarakhand	2014	113	4	10	14	12.39	99	64	82.05	3.54	8.85	9.39	17.17	Shah et al. [29]
District Garhwal North West Himalaya	1999	2035	19	45	64	3.14	1971	14	17.95	0.93	2.21	3.33	6.44	Gaur [11]
Kumaon Himalaya India	2014	89	8	14	22	24.72	67	56	71.79	8.99	15.73	21.78	35.77	Singh et al. [27]
Central Himalaya India	2002	50	3	2	5	10.00	45	73	93.59	6.00	4.00	4.42	8.47	Negi et al. [18]
Jammu Kashmir and Ladakh India	2014	948	25	15	40	4.22	908	38	48.72	2.64	1.58	4.41	8.45	Gairola et al. [44]
Kashmir Himalaya	2011	30	5	7	12	40.00	18	66	84.62	16.67	23.33	16.66	28.57	Malik et al. [59].
Himachal Pradesh North west Himalaya, India	2016	73	11	8	19	26.03	54	59	75.64	15.07	10.96	20.21	33.62	Thakur et al. [28].
Himachal Pradesh North west Himalaya, India	2007	643	7	12	19	2.95	624	59	75.64	1.09	1.87	2.86	5.56	Samant et al. [36]
Nepal Himalaya	2006	84	3	5	8	9.52	76	70	89.74	3.57	5.95	5.79	10.95	Kunwar et al. [37]
Arunachal Pradesh Eastern Himalayan zone	2011	74	6	10	16	21.62	58	62	79.49	8.11	13.51	15.38	26.66	Tangjang et al. [17]
Average		172.14	7.69	10.23	17.91	21.68	154.23	60.09	77.03	9.14	12.54	15.49	25.11	

This occurrence may be due to the sharing of a similar flora and the cross-cultural exchange of medicinal plant knowledge in past and present. It also indicates similar ethno-genesis of people in comparative areas [47]. Besides, low similarity indices may be likely due to minimal cultural exchange between the mountains region as they are disconnected through mountain ranges and other cultural variations [24]. However, region to region similar medicinal flora are used in various way. Low similarity with the other report may be due to different topography and climatic condition and medicinal flora or it could be a sign of loss of cultural practices.

### Novelty and future prospects

The present study was compared with the previous studies related to analysis of ethnomedicinal plants and their uses in Himalaya. This comparative analysis in the ethnomedicinal point of view found the following new reports as *Calotropis gigantea* for joint pain, swelling (37 UR) and skin diseases (2 UR); *Citrus aurantiifolia* for dysentery, diarrhea and as refrigerant with 42 UR; *Cucumis sativus* for fever with 65 UR; *Dioscorea bulbifera* for fever (17 UR) and boils (16 UR); *Drymaria cordata* for herpes (6 UR) fever and headache (13 UR); *Duchesnea indica* for Skin diseases (12 UR) and as refrigerant (14 UR); *Engelhardtia spicata* for cleansing teeth (37) and treatments of boils, cut and wounds (50 UR); *Hedychium spicatum* for skin diseases and boils, cut and wounds, joint pain (26 UR); *Hordeum vulgare* for weakness (9 UR) as refrigerant (17 UR); *Mangifera indica* used for stomachache (12 UR), dysentery and diarrhea (19 UR) (especially for child); *Prunus persica* used for boils, skin diseases (12 UR) and as refrigerant (30 UR); *Polygonum capitatum* for boils, burnt (21) herpes (1); *Pouzolzia hirta* to remove dandruff and prevent hair fall (92 UR); *Rubus ellipticus* for throat infection (17 UR), boils and skin diseases (9 UR) and cleaning teeth (26 UR); *Stephania elegans* for headache (4 UR), acts as refrigerant (4 UR), fever (4 UR); *Smilax aspera* for snake-bite and scorpion-sting (2 UR), *Taxus wallichiana* for boils (27 UR), cuts and wounds (15 UR) and *Trichosanthes tricuspidata* for fever (65 UR) (Table 3) were newly reported ethnomedicinal uses.

**Fig. 11:**
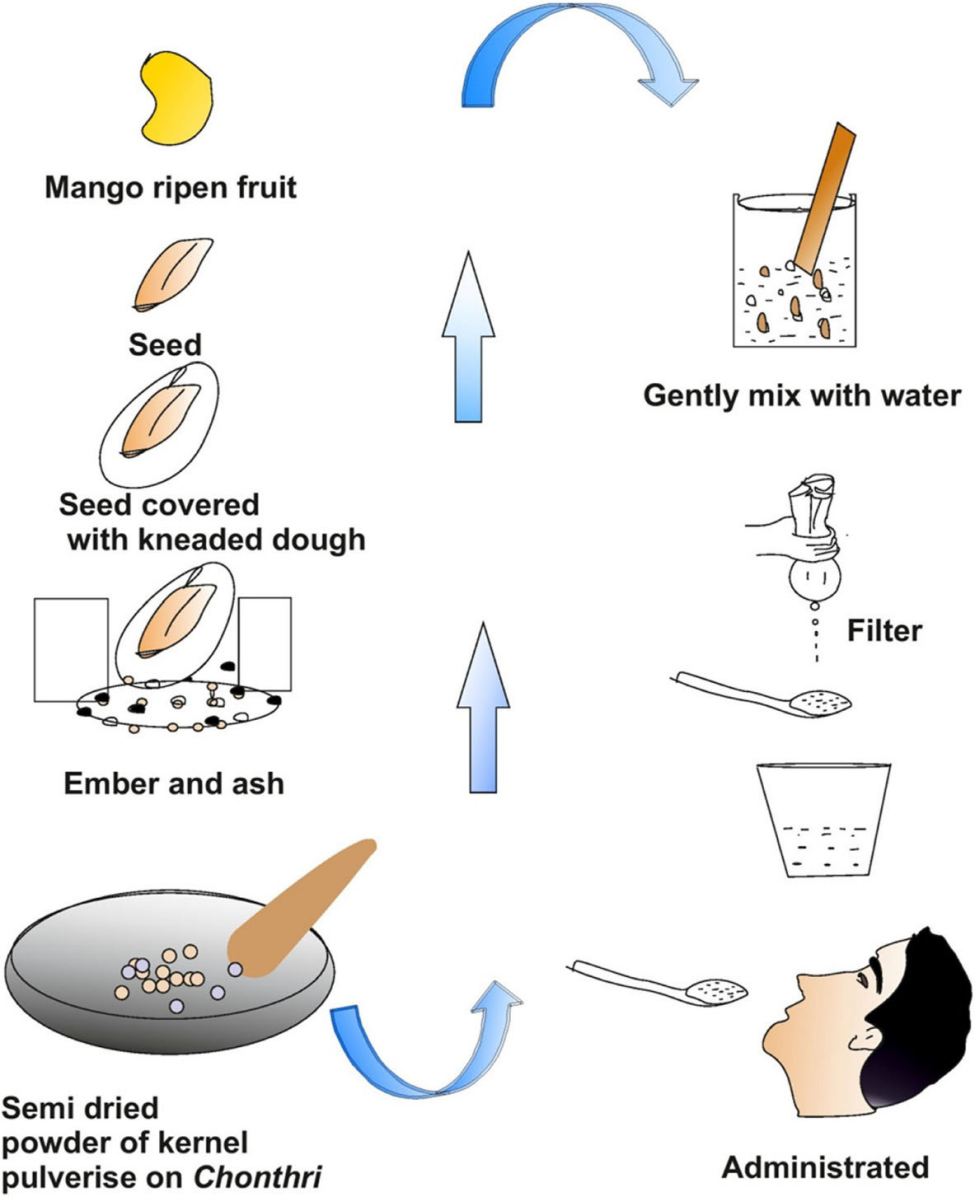
Preparation of seed extract by local inhabitants of Jakholi

Some of plant species such as *Aconitum heterophyllum*, *Eupatorium adenophora*, *Echinochloa frumentacea*, *Engelhardtia spicata, Megacarpaea polyandra*, *Picrorhiza kurroa*, *Polygonum capitatum*, *Plantago depressa*, *Potentilla fulgens*, *Quercus leucotrichophora*, *Senecio nudicaulis* were frequently used in Jakholi but their detailed bioactive constituents and pharmacological activity are yet unknown, revealing a good candidature for pharmacological and therapeutic values and extraction of novel bioactive constituents (Fig. 11).

## Conclusions

Present paper is the first attempt of survey in Jakholi Block, Uttarakhand, India. Asteraceae, Polygonaceae, Ranunculaceae and Rosaceae were the most used families and root were the most commonly used plant parts in the area. *Aconitum heterophyllum*, *Megacarpaea polyandra*, *Picrorhiza kurroa* and *Rheum emodii* are well known medicinal plant species, contributing important role in the local health care system of Jakholi area. Documentation of local medicinal knowledge is also essential due to outmigration of the younger. Study of ethnomedicinal knowledge helps identify the important species of the region for pharmacological importance and ecological sustainability and it also aids conservation of traditional knowledge. Cataloguing useful plant species supports registration of indigenous knowledge, aiding national impetus of obeying implementation of convention of biological diversity and Nagoya protocol. Traditional knowledge is based on experience passed on from generation to generation and limited only to elderly (*Bujurg*) people and traditional healers. We came to the following considerations to be taken while doing ethnomedicinal studies in the Himalaya: (a) local people are quite conservative in sharing traditional knowledge about the Medicinal plants; (b) the young generation is not interested and knowledgeable about the ethnomedicinal plants and their uses; and (c) outmigration is a menace to the conservation of traditional ethnomedicinal knowledge. The present study showed that the medicinal plants are still very important for livelihood of local inhabitants of Jakholi and the Himalaya. Some medicinal plants are at the brisk of threatened due to their ecology, biology and human induced exploitations. To sum, documentation of useful plants and the knowledge of their utilization is immediate before being lost.
